# Synthesis of Hollow Pt-Ni Nanoboxes for Highly Efficient Methanol Oxidation

**DOI:** 10.1038/s41598-019-51780-y

**Published:** 2019-10-24

**Authors:** Rabia Jamil, Manzar Sohail, Nadeem Baig, Muhammad S. Ansari, Riaz Ahmed

**Affiliations:** 1 Department of Chemistry, School of Natural Sciences, National University of Science and Technology (NUST), Islamabad, 44000 Pakistan; 2 Chemistry Department, King Fahd University of Petroleum and Minerals, Dhahran, 31261 Saudi Arabia; 3 Department of Chemistry, Quaid-i-Azam University, Islamabad, 45320 Pakistan

**Keywords:** Electrocatalysis, Fuel cells

## Abstract

In direct methanol fuel cell technology, highly stable electrochemical catalysts are critically important for their practical utilization at the commercial scale. In this study, sub ~10 nm hollow Pt-Ni (1:1 at. ratio) nanoboxes supported on functionalized Vulcan carbon (Pt-Ni/C-R2) were synthesized through a facile method for the efficient electrooxidation of methanol. Two reaction procedures, namely, a simultaneous reduction and a modified sequential reduction method using a reverse microemulsion (RME) method, were adopted to synthesize solid Pt-Ni NPs and hollow nanoboxes, respectively. To correlate the alloy composition and surface structure with the enhanced catalytic activity, the results were compared with the nanocatalyst synthesized using a conventional NaBH_4_ reduction method. The calculated electroactive surface area for the Pt-Ni/C-R2 nanoboxes was 190.8 m^2^.g^−1^, which is significantly higher compared to that of the Pt-Ni nanocatalyst (96.4 m^2^.g^−1^) synthesized by a conventional reduction method. Hollow nanoboxes showed 34% and 44% increases in mass activity and rate of methanol oxidation reaction, respectively, compared to solid NPs. These results support the nanoreactor confinement effect of the hollow nanoboxes. The experimental results were supported by Density Functional Theory (DFT) studies, which revealed that the lowest CO poisoning of the Pt_1_Ni_1_ catalyst among all Pt_m_-Ni_n_ mixing ratios may account for the enhanced methanol oxidation. The synthesized hollow Pt-Ni/C (R2) nanoboxes may prove to be a valuable and highly efficient catalysts for the electrochemical oxidation of methanol due to their low cost, numerous catalytically active sites, low carbon monoxide poisoning, large electroactive surface area and long-term stability.

## Introduction

The global focus on clean and sustainable energy sources has been urgently prompted by environmental pollution, fear of depletion of fossil fuels and rapid climatic changes^1^. Fuel cell technologies have attracted significant attention for power generation due to their portability, high energy conversion efficiency, and environmental friendliness^2^. For most practical applications, the hydrogen-oxygen fuel cell has displayed the capability to provide efficient energy. However, in fuel cells, the usage of hydrogen is still a serious problem due to the issues related to its distribution, storage, and production. Liquid fuels, such as methanol and ethanol, are promising alternatives in terms of their storage and distribution^2^.

Among fuel cells, both direct methanol fuel cells (DMFCs) and direct ethanol fuel cells (DEFCs) are drawing great attention due to their simple structures^3^, high efficiency, low pollution, low operating temperature, and good availability^4^. DMFCs have few advantages over DEFCs such as low CO_2_ emissions and no need for a catalyst to break C-C bond in ethanol. Furthermore, use of ethanol as a fuel legally requires denaturalization by using ethers which decrease the efficiency. DEFCs so far have been demonstrated only at micro-scale. Methanol can easily be synthesized from the natural gas as well as from renewable biomass. It can be easily transported and safely stored^5–7^. The performance of the DMFCs depends on how efficiently methanol oxidizes on the anode surface. One of the challenges in methanol oxidation at the anode is its high overpotential. Therefore, there is high demand for an efficient catalyst that can facilitate the electrooxidation reaction of methanol at the electrode surface. As a key constituent of fuel cells, nanocatalysts having controllable compositions, structures, and properties have always been required to get desired results. In this regard the conversion of chemical energy into electricity in direct methanol fuel cells (DMFCs) requires the synthesis of catalysts to achieve better performance in terms of efficiency and durability.

Platinum is proved the most efficient catalyst for the electrooxidation of the methanol. However, the pure platinum catalyst has some inherent drawbacks, such as its high cost, limited reservoirs, and poor tolerance of intermediate species, such as CO, which drastically affect the oxidation reaction of methanol at its surface. In the widespread use of fuel cell technology, cost is one of the major barriers due to the high cost of the Pt metal catalyst. To overcome the challenges of stability and reduce the cost of the Pt catalyst, many serious efforts have been made for methanol oxidation reaction^8^. The activity and stability of platinum were improved by tailoring the heterostructures with non-precious metals, such as Ni, Co, Fe, and Cu, for fuel cells^9–13^. Ni-based materials are good electrocatalysts due to their enhanced catalytic properties and low CO poisoning during the electrooxidation of methanol^1,14^. Over the last few years, the poising tolerance and the activities of the Pt-based catalyst were improved by introducing various shapes of nanostructures including nanowires^15,16^, nanoparticles^17^, mesoporous^18^, nanospheres^19,20^, and nanotubes^21,22^. Hollow metallic nanoarchitectures are an interesting class of materials due to their unique physical and chemical properties^23^. Hollow nanostructures are considered an extraordinary morphology due to their ‘nanoreactor confinement effect’. The hollow or porous networks provide low density, large surface area, low cost and unique electronic characteristics^24^. In solid nanostructures, only the exterior part is available for catalytic activity, while the interior atoms do not playing considerable catalytic role. In addition to nanoreactor confinement, hollow metallic nanostructure expose more interior atoms for catalytic activity^25–28^. Further, more edges and corners are available compared to solid nanostructures. Various Pt-containing mono, di, and trimetallic hollow nanostructures were introduced for the electrochemical oxidation of methanol^19,29,30^. Their large surface area, low density and high void ratio present a new era of nanocatalysis.

In general, hollow nanostructures can be fabricated by sacrificial templates via the Kirkendall effect, galvanic replacement, selective chemical etching and so on^31–34^. Among different methods, the sacrificial technique has been extensively used to fabricate hollow nanostructures. The template-assisted process faces few challenges including high cost, imparting impurities and tedious synthesis^35^. It is not easy to dissolve hard templates without damaging the newly prepared nanostructured shells. The microemulsion is considered a soft template technique and is fascinating for the fabrication of massive scale and hollow nanostructures due to its simplicity and tunability for the size and composition of the nanostructures^36^. A water in oil emulsion is called a reverse microemulsion and assists to attain ultrafine metal nanoparticles with a controlled and uniform size^37^. The size, distribution and morphology of the NPs have a substantial effect on the material’s catalytic behavior. In this regard, the reverse microemulsion helps to achieve better size control and phase purity^38–40^. In previous literature, the sequential reduction method was frequently used to synthesize core-shell NPs^41–44^.

In the present work, Pt-Ni NPs and hollow nanoboxes were uniformly deposited on the functionalized carbon support using reverse microemulsion (RME). Ni was selected as a co-metal with Pt due to its low cost and comparatively better catalytic behavior. The simultaneous reduction method and sequential reduction method were adopted to synthesize NPs and hollow nanoboxes, respectively. Here, we report an RME method with slight modification for the fabrication of hollow nanocubic Pt-Ni structures. The catalytic activity of the newly synthesized Pt-Ni/C nanoboxes was compared with Pt-Ni/C nanostructures obtained by a conventional NaBH_4_ reduction method. The structure-activity relationship and catalytic activity were rationally elaborated by theoretical analysis.

## Experimental

### Materials and chemicals

All chemicals were of analytical grade and purchased from Sigma-Aldrich, used without further purification.

Deionized water (resistivity 18.2 MΩ.cm at 25 °C) was used for aqueous solutions’ preparation.

### Pretreatment of vulcan carbon (VC)

Purification and functionalization of VC with oxygenated functional groups was performed as described elsewhere^45,46^. Briefly, 0.2 g of VC was purified by acid treatment in a round bottom flask containing 100 mL of 6 M HCl. The contents were refluxed for 4 hours at 60 °C. After refluxing, acid-treated VC was washed several times with de-ionized water until the filtrate had a neutral pH. The purified VC was vacuum-dried for 24 hours at 110 °C. The purified VC was subjected to functionalization using a mixture of 8 M HNO_3_ and 35% H_2_O_2_ (4:1 v/v). The remaining steps were the same as described for purification.

### Synthesis of Pt-Ni/C through conventional reduction (CR) method

The Pt-Ni/C was synthesized and supported on the VC through a conventional co-reduction method under an argon atmosphere at room temperature using NaBH_4_ as a reducing agent. In detail, 4 mL of an aqueous solution of 0.03 M PtCl_4_ and 1.4 mL of an aqueous solution of 0.07 M NiCl_2_.6H_2_O along with 0.2 g of VC were added to a three-neck round bottom flask containing 50 mL of de-ionized water. The golden-colored mixture was stirred under an argon atmosphere until it became colorless due to metal ion adsorption on the VC. Then, 5 mL of the freshly prepared with 0.05 g of NaBH_4_ was added dropwise and stirred continuously for 4 hours, which resulted in a black suspension. The suspension was filtered and washed until a neutral pH was obtained and was vacuum-dried at 110 °C for 24 hours. The Pt-Ni/C synthesized by this method was labeled CR.

### Synthesis of Pt-Ni/C with RME method

The Pt-Ni NPs and nanoboxes were synthesized by simultaneous addition and sequential addition RME methods, respectively^8,11,12^. The simultaneous addition RME method is a single-step process leading to homogeneous alloy formation, and the modified sequential addition RME method produced hollow nanoboxes of Pt-Ni/C. The RME system was composed of Triton X-100 as the surfactant, *n*-hexanol as a co-surfactant and the Pt-Ni precursor solution and/or a NaBH_4_ solution dispersed in a continuous oil phase of hexane.

### Simultaneous reduction method

The simultaneous reduction RME method is a two-emulsion technique that was employed for the synthesis of Pt-Ni/C labeled R0. In this method, the 12.5 mL of TX-100, 25 mL of hexane, 25 mL of *n*-hexanol, 3 mL of 0.03 M PtCl_4_ and 1.02 mL of 0.07 M NiCl_2_.6H_2_O solution (Table S1) were added into a three-neck flask reactor under argon. The mixture was stirred at room temperature for 30 min to form RME (I). After this treatment, the solution color appeared pale yellow due to the mixed color of the Pt and Ni ions. RME (II) contained 4 mL of 1.4 M NaBH_4_ as an aqueous phase and was prepared by mixing all the components, as depicted in Table S2. RME (II) was colorless due to the absence of metallic ions. The Pt-Ni/C-R0 NPs were attained by mixing RME (II) and RME (I). The addition of RME (II) into RME (I) turned the color of the solution from pale yellow to dark due to the reduction of the PtCl_4_-NiCl_2_.6H_2_O solution by a NaBH_4_ reducing agent. The mixture was stirred for another 2 hours under an argon atmosphere.

### Modified sequential reduction method

Catalysts labeled R1 and R2 were synthesized by the sequential reduction method, which was a four-emulsion technique (Table S2). RME (I) and RME (II) were prepared and added similar to simultaneous reduction process and stirred for 1 h. Next, RME (III) (which was stirred for 30 min before adding to reaction mixture) was added to the reaction mixture and stirred for 0.5 h or 1 h (depending upon the catalysts) at room temperature. The catalysts were labeled R1 and R2 when the mixing time was 0.5 h and 1 h, respectively, and was the only difference among R1 and R2, which proved to be a crucial factor in the appearance of Pt-Ni nanoboxes. Finally, RME (IV) was transferred into the abovementioned three-neck flask reactor under Ar gas and stirred at room temperature for 2.5 h.

### Adsorption of NPs on VC

In the RME solutions (both simultaneous and sequential procedures) containing Pt-Ni NPs, 0.15 g of purified functionalized VC was added and stirred overnight. The reverse micelles were broken by adding 50 mL of ethanol, and stirring was continued for another 30 minutes. The precipitate was filtered using a 0.22 µm Millipore filter. The filtered precipitate was extensively washed with 1:1 water/ethanol and hexane/acetone. After washing, the product was vacuum-dried overnight at 160 °C. Simultaneous and sequential procedures are represented in Figs S1 and S2.

### Electrochemical studies

A conventional three electrode system attached to EcoChemie Autolab PGSTAT 12 potentiostat/galvanostat was used for all electrochemical measurements. For working electrode fabrication nanocomposite materials were drop casted over a graphitic electrode with a surface area of 0.785 cm^2^. A saturated calomel reference purchased from Fisher Scientific Company was used as a reference while Platinum wire gauze was used as a counter electrode. For polishing of the working electrode 1.0 μm alumina (CHI Inc., USA) was used and then electrode surface was sonicated in ethanol and ultrapure water respectively to remove any impurities from its surface. The solution for drop casting was prepared by making suspension of 2 mg of catalyst in 1 mL mixture of water and iso-propanol. Next, 45 μL of the catalyst suspension was drop casted over the graphitic working electrode and air dried. Once solvent was evaporated, 10 μL of binder solution (5% Nafion) was added and again air-dried^47^.

### Computational details

The Vienna ab initio simulation package (VASP), was used for performing an iterative solution of Kohn-Sham equations on a plane-wave basis set. The conditions used are described elsewhere^48^. Briefly, calculations were performed by including plane-waves with a kinetic energy below or equal to 400 eV. A projector-augmented wave (PAW) method, which essentially combines the accuracy of all-electron methods and the computational simplicity of the pseudopotential approach^49^, was used to describe the electron-ion interaction for Pt, Ni, C and O atoms^50^. For CO molecule was calculations a cubic unit cell of 10 × 10 × 10 Å^3^ was used. The calculated binding energy of gas-phase CO was E_CO(gas)_ = −14.82 eV. The calculated CO bond length was in the range of 1.14–1.19 Å, very closely matching with the experimental value of 1.15 ± 0.05 Å^51^. The five-metal layer slan model approximation was used for the Pt (111) and Ni (111) surfaces. The unit cell chosen was a p (2 × 2) for the surface coverage of ¼ ML. The adsorption energy was calculated using Eq. 1.

1 $${{\rm{E}}}_{{\rm{ads}}}={{\rm{E}}}_{{\rm{CO}}/{\rm{M}}}-{{\rm{E}}}_{{\rm{M}}}-{{\rm{E}}}_{{\rm{CO}}({\rm{gas}})}$$

## Results and Discussion

### Density functional theory (DFT) studies

**Figure 1:**
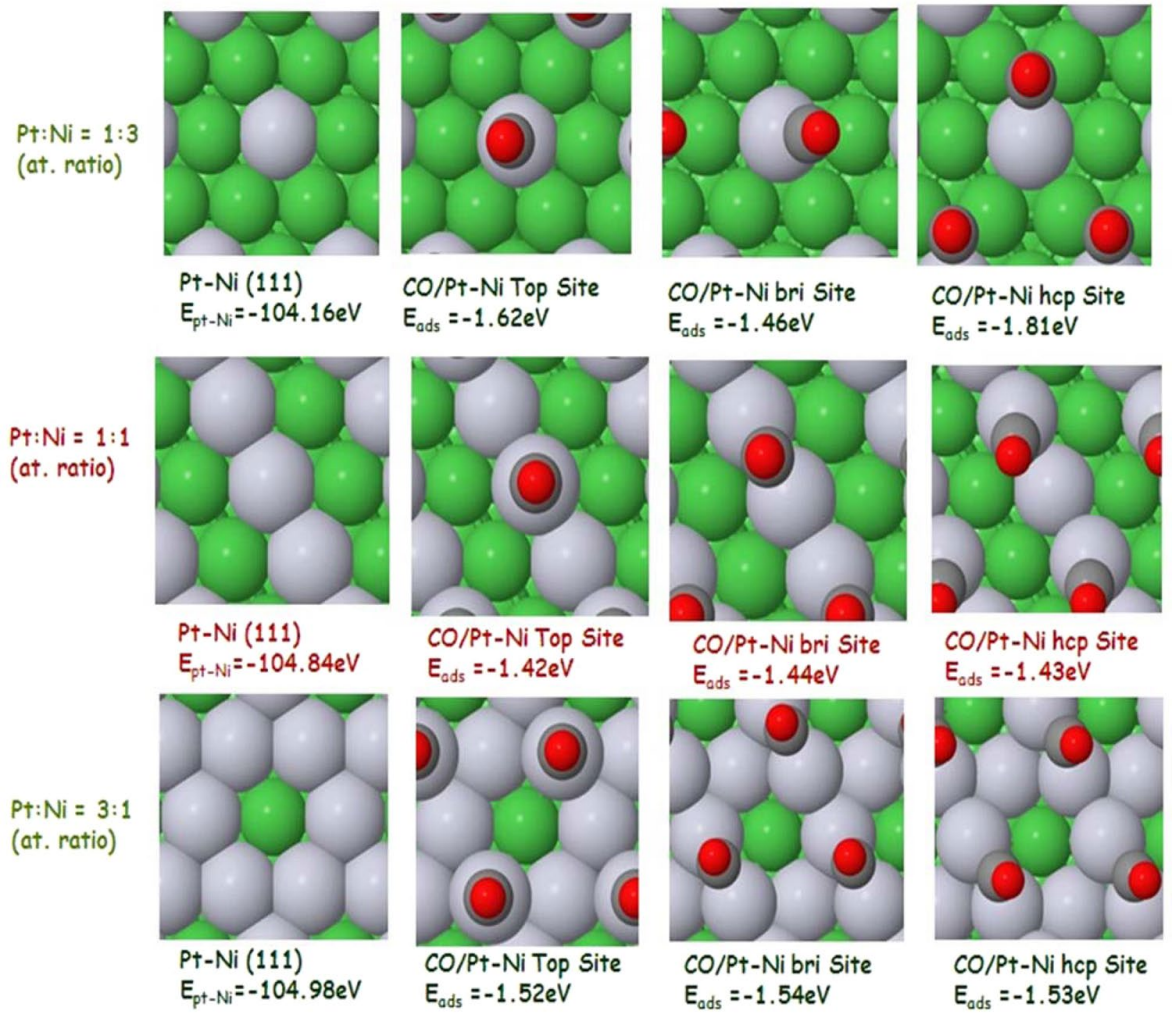
Overview of the effect of the mixing ratio on the E_ads_ of CO on Ptm-Nin alloy (green atoms = Ni, grey atoms = Pt).

To investigate the promoting effect of Ni to Pt in bimetallic catalysts for enhanced MO, DFT studies of the CO adsorption were performed on Pt and Pt_m_Ni_n_ models. In the DFT analysis, CO was the focus instead of methanol adsorption for two reasons. First, CO is the main poisoning species, and second, CO oxidative removal is the rate-determining step in the MO reaction. Hence, the study of the CO adsorption properties proved to help clarify the catalyst mechanism. The slab model approximation approach was chosen to describe the structure of bimetallic catalysts. Although this approximation may not be fully representative of the actual solid surface of Pt-Ni/C, it performs DFT calculations that provide a suitable understanding towards reaction mechanism. First, full geometric optimizations were executed on Pt, Ni, and Pt_m_Ni_n_ prior to the adsorption studies. XRD patterns of the bimetallic reveal that the maximum shift in the 2-theta position is observed for the Pt (111) surface. Therefore, the Pt (111) surface was chosen for the adsorption studies. Pt has an fcc structure, and this structure is enhanced by alloying with Ni, as shown in Fig. 1.

For comparison, pure Pt and Ni surfaces were analyzed together with Pt_m_Ni_n_ bimetallic alloy. The CO adsorption strength on the surface of the metal depends on the adsorption sites, configuration, and composition of the slab. Therefore, three CO adsorption sites i.e., a top site, bridge site, and hcp/fcc sites, on the Pt, Ni and Pt_m_Ni_n_ slabs were investigated. For the pure Pt slab, the CO adsorption energy on the hcp, bridge and top sites follows a decreasing order, as shown in Fig. S3. The E_ads_ values for the hcp, bridge and top sites on pure Pt were −1.64, −1.59 and −1.48 eV, respectively, indicating that the adsorption on the hcp site in pure Pt is dominant. The CO adsorption on a pure Ni slab follows the same trend, as shown in the Table S3. CO adsorbs more strongly on all three sites for Ni compared to those of the Pt surface, and the CO bond stretches more on Ni surface. Different adsorption sites for Ni are shown in Fig. S4. The slab consisting of top two layers of Pt followed by a third layer of Ni atoms is shown in Fig. S5. From Table S3, it is obvious that the E_ads_ for the three sites is much lower than those of Ni but slightly higher than those of Pt, showing that a charge transfer phenomenon that modified the electronic structure as well as surface chemical properties of Pt has occurred. This model can be correlated with the Ni@Pt core-shell structure, which still modifies the properties of surface Pt even though Ni is present in the core. A comparative study of the bimetallic mixed alloy, as shown in Fig. 1, with different atomic ratios of Pt and Ni was evaluated to elucidate the effect of the mixing ratio on the CO adsorption. Three combinations were selected for this study, i.e., Pt_1_Ni_3_, Pt_1_Ni_1,_ and Pt_3_Ni_1_. In the experimental investigations, Pt_1_Ni_1_ was used. In 5 layered slab models, doping occurred on the top layer while the remaining layers consisted of Ni. Different adsorption sites for each combination were displayed in Fig. 1. It is interesting to find that E_ads_ for the three sites of Pt_1_Ni_1_ was minimum among the three combinations and was lower than those of pure Pt. This theoretical observation complements the reported experimental work. It can be suggested that in the case of Pt_1_Ni_1_, an equal number of Ni and Pt atoms were present, thus producing minimal CO poisoning. When more Ni atoms were present than Pt, i.e., Pt_1_Ni_3,_ the E_ads_ was the highest among the three combinations, and thus, CO poisoning was the highest of the three adsorption sites. The same trend was observed for Pt_3_Ni_1_; however, the CO poisoning was lower compared to Pt_1_Ni_3_. Based on these observations, it can be inferred that the MO activity is sensitive to the mixing ratio of Pt with Ni, and the maximum activity can be achieved when an equal number of Pt and Ni atoms are present. The electronic structure of Pt is modified by charge transfer from Ni to Pt, which increases the electron density of the Pt d orbital, weakening the interaction between the Pt_1_Ni_1_ surface and adsorbed CO and may therefore result in decreased CO poisoning and increased MO activity^52^.

### Structural and morphological analysis

Pure VC is non-polar in nature and consists of a sp^2^ hybridized structure (C=C bond). The different oxygen-containing functionalities, such as carbonyl and hydroxyl groups, were generated on the surface of VC during its pretreatment with 8 M HNO_3_ and 35% H_2_O_2_. The FTIR spectra of the acid-functionalized VC revealed the presence of oxygen functionalities (Fig. S6). The absorption bands that appeared at 1601 and 1380 cm^−1^ were attributed to sp^2^-hybridized C=C stretching vibrations. The absorption bands observed at 3620 cm^−1^ and 1719 cm^−1^ were assigned to –OH and C=O, respectively. The introduction of the oxygen-containing functional groups enhanced the surface polarity of VC. The presence of these functional groups on the surface of VC improved the electrochemical surface area and facilitated the dispersion and loading of the catalyst^53^. Apart from this, the presence of the polar functionalities on VC also improved the dispersibility of the catalyst in aqueous and organic media, which is a highly desired property for the fabrication of electrodes and nanocomposites^54–56^.

**Figure 2:**
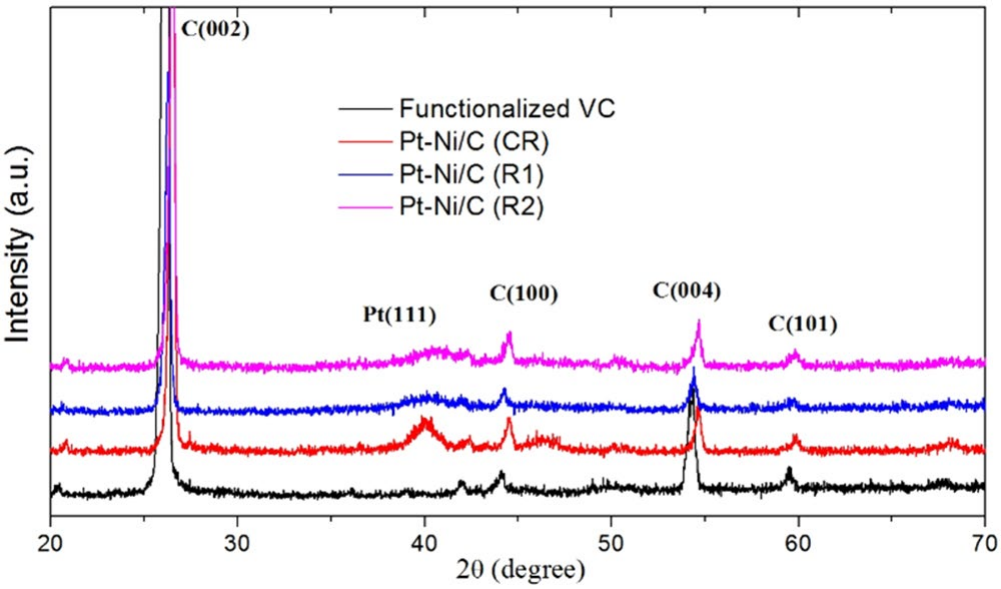
XRD patterns of VC, Pt-Ni/C (CR) and Pt-Ni/C (RME).

Figure 2 reveals the XRD patterns of purified functionalized VC and Pt-Ni/C synthesized through the CR and RME methods. The strong diffraction peak appearing in the region of 20 to 30° is generally attributed to VC [39]. The peaks located at the 2θ positions of 26.19°, 44.14°, 54.39° and 59.54° are associated with the (002), (100), (004) and (101) planes of the graphite-like structure of VC^57–59^. Purified functionalized VC was used as a support for the synthesis of nanocatalysts. The XRD pattern of purified functionalized VC displayed no extra peaks related to metal or metal oxide impurities. Generally, the XRD spectrum of crystalline Pt exhibits four characteristic peaks corresponding to the (111), (200), (220), and (311) planes [40]. In the XRD spectra of different Pt-Ni/C composites, the (111) plane was clearly observed, while the rest of the planes were difficult to observe due to their poor intensity. The XRD pattern displayed the fcc crystallinity of the Pt-based synthesized catalysts.

In the XRD spectra of the various synthesized Pt-Ni catalyst, no individual peak for Ni or its oxides/hydroxides were observed, which might be due to partial alloy formation/incorporation of Ni (alloying element) in the Pt structure^60,61^. However, a shift in the Pt (111) peak with the incorporation of Ni was observed depending on the degree of alloying. The peak of Pt (111) plane generally appeared at 39.67°. The incorporation of Ni showed a significant effect on the Pt (111) plane, and the peak was regularly shifted from 39.67° to 40.07°, 40.52°, and 40.82° for CR, R1 and R2, respectively. The decrease in the Pt (111) peak intensity and its broadening were also observed when the peak shifted towards higher 2θ values^62^. These XRD results revealed the maximum incorporation of the alloying element in the Pt (111) plane, which caused strain and distortion in the crystal structure. The evaluated XRD parameters demonstrate that there is a significant difference in the Pt-Ni/C synthesized by the CR or RME methods (Table S4). For the confirmation of the alloy formation, the XRD pattern of bimetallic nanocatalysts was compared with the Pt standard pattern (00-001-1190). A significant decrease in the d-spacing, lattice parameters, cell volume, and Pt-Pt bond length and an increase in the X-ray density were found, which confirmed the alloy formation of Pt with Ni. Measuring the change in the lattice constant during alloying is a convenient approach for expressing the extent of alloying^63–66^. A significant decrease in the lattice parameters of the RME-synthesized nanocatalyst (3.829 Å) was observed compared to the CR method (3.897 Å). These results revealed the higher degree of alloying during the RME synthesis. As during RME synthesis, flexible surfactant film (TX-100) allows greater intermicellar exchange thus enabling small size Ni atoms to significantly replace the Pt atoms to form the Pt-Ni alloy^62^. The Debye-Scherrer equation was applied to obtain a rough estimate of the average crystallite size for CR- and RME-synthesized Pt-Ni/C. The RME method significantly decreased the size from 14.3 nm (CR method) to 5.1 & 4.5 nm. These results demonstrate that the RME method was more efficient for the preparation of the nanocatalyst.

**Figure 3:**
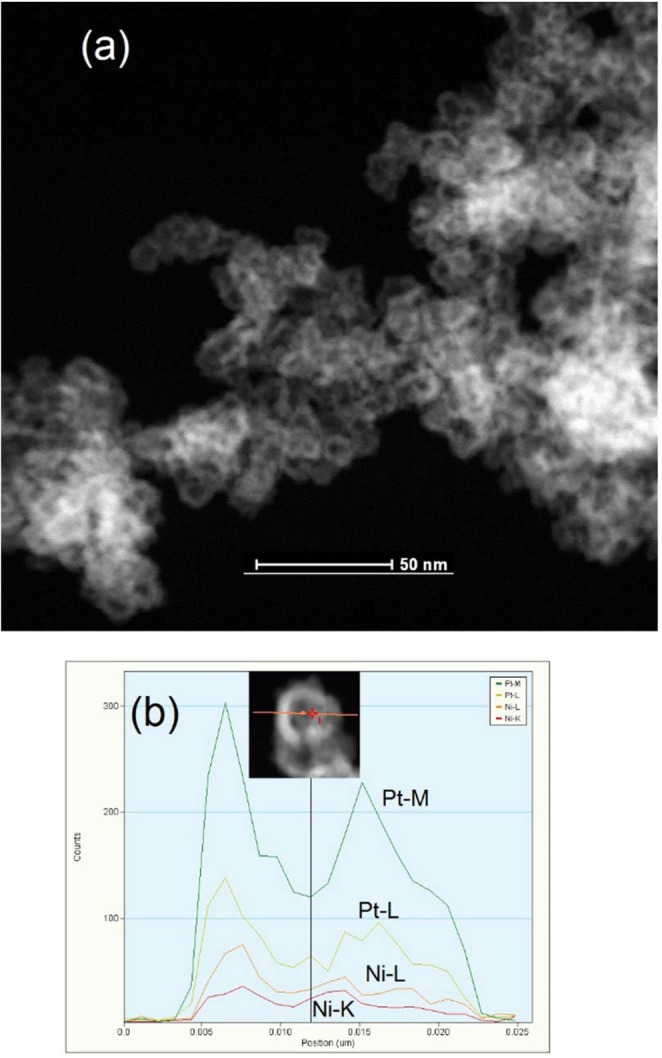
STEM-HAADF image at (**a**) 50 nm and (**b**) EDX line scan along the arrow of Pt-Ni/C(R2) (inset- HAADF-STEM of a single hollow nanobox).

Figure 3 shows the high angle annular dark field (HAADF)-scanning transmission electron microscope (STEM) images of R2. Since the average hydrodynamic radius of the RME was 17.1 nm^67^, R0 and R1 showed NPs in the range of 3–5 nm and nanoboxes in the range of 5–10 nm. Compared to the sequential reduction method, the simultaneous reduction method generated spherical, small and solid NPs (Fig. S7B). Moreover, the sequential reduction method can be modified to obtain both hollow nanoboxes (Fig. 3) and solid NPs (Fig. S7C). To further elucidate the structure and chemical composition of nanoboxes and solid NPs, energy dispersive X-ray (EDX) analysis with individual nanoboxes/solid NPs was conducted. Figure 3b shows the EDX line scan along the red line at the HAADF-STEM of a single hollow nanobox (inset). HAADF-STEM image and a corresponding EDX line scan show the Pt and Ni elemental distribution (Fig. 3(b)). The sharp difference in the contrast between the center and edge in the HAADF-STEM image indicates the structure was hollow, an observation that is further supported by the saddle-type shape of the Pt M-line and Ni L-line curve with the weakest signal in the center region^28^ compared to the bell-shaped curves of CR, R0, R1 presented in the Figs S9, S10 and S11. In the sequential reduction, the mixing time before the addition of RME (IV) was important for controlling the characteristics of the material. As mentioned in the experimental section, the R1and R2 catalysts were synthesized by varying this mixing time. In general, a minimal number of atoms come together to form a stable nucleus at the start, followed by collisions between these groups of atoms, thus leading to the assembly of stable nanoparticles. There is a possibility that once the nuclei are formed, the growth process may surpass the nucleation. Interruption of the process generated exceptional results, which are evident from the synthesis of the different nanocatalysts R1and R2 (Fig. S2). If the mixing time was less than 1 h, then solid NPs formed (Fig. S7C). No metal segregation is observed by using flexible surfactant (TX-100) even in case of sequential reduction method which is in accordance with the study of C. Tojo^68,69^. The Table S5 shows the EDX analysis of the Pt-Ni/C nanocatalysts synthesized through two methods. The EDX spectra displayed the presence of the Pt and Ni in the catalysts without any significant impurities. The catalysts synthesized through the RME methods displayed a mass ratio and atomic ratios close to the theoretical values (Table S5). Moreover, Pt loading was also close to the actual mixing ratio. The uniform dispersion of Pt not only improves the performance of the catalyst but also lowers on the cost of the material. The mapping study demonstrated the uniform distribution of the Pt-Ni catalyst over VC through the RME method compared to the conventional method in all the prepared catalysts (Fig. S8).

### Electrochemical characterization of the conventional and RME method Synthesized catalysts

**Figure 4:**
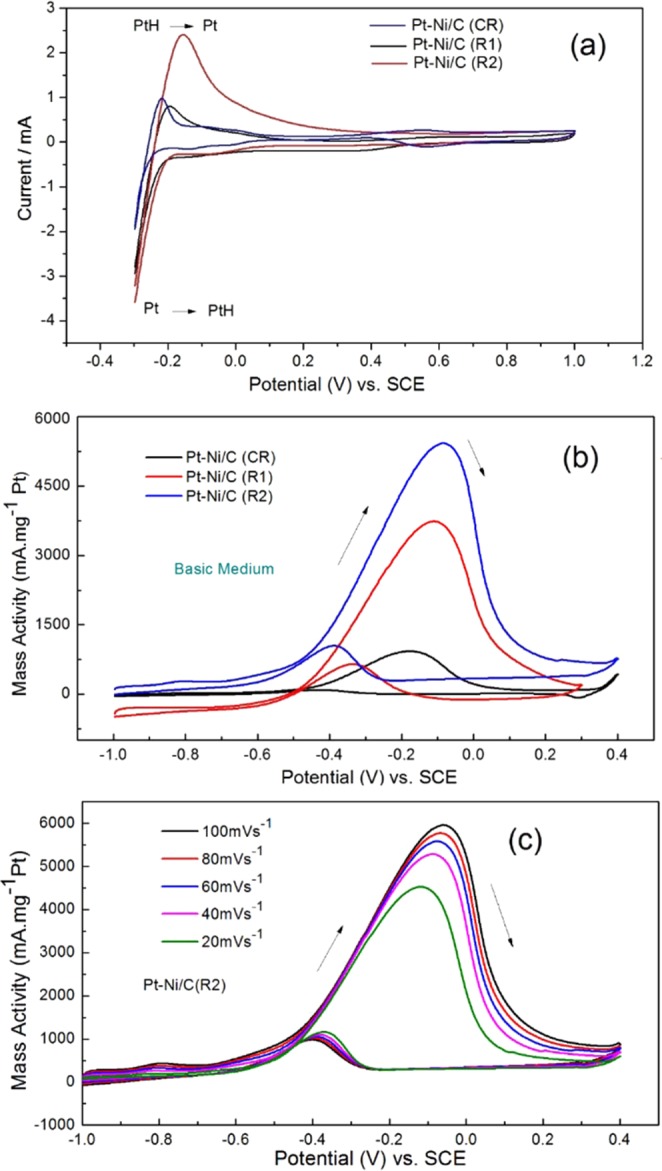
(**a**) CVs measured in 1 M H_2_SO_4_ (**b**) CVs measured in 1 M CH3OH + 1 M KOH at scan rate 50 mV.s^−1^ on Pt-Ni/C (CR) and Pt-Ni/C (RME) catalysts. (**c**) CVs measured in 1 M CH_3_OH + 1 M KOH at different scan rates on Pt-Ni/C-R2.

The electrochemical activity of the different synthesized catalysts was evaluated by scanning their cyclic voltammetry (CV). Figure 4a shows the characteristic CVs of CR, R1 and R2 recorded in 1 M H_2_SO_4_ at a scan rate of 50 mV s^−1^ in the potential range −0.3 to 1.0 V vs. SCE. In the forward scans, the oxidation of hydrogen occurs from −0.25 to 0.05 V and represents the desorption of hydrogen at the catalyst surface, while in the reverse scan, the reduction of hydrogen occurs from 0.05 to −0.25 V and represents the adsorption of hydrogen on the catalyst surface^70,71^. The catalytic activity of the synthesized catalysts in terms of electrochemical surface area (S_ESA_) is evaluated by using Eq. 2.

2 $${\rm{SESA}}={\rm{Qct}}/{\rm{QPt}}.{\rm{L}}$$

where Q_ct_ is the charge transfer from the catalyst in hydrogen adsorption region (µC), Q_Pt_ is the charge transfer for monolayer hydrogen adsorption (210 µC cm^−2^) on an ideal polycrystalline Pt surface (Pt–H = 1:1), and L is the mass of Pt loading (mg)^72^. The electrochemical surface area of the hollow nanoboxes was found to be significantly higher, i.e., 2 and 1.6 times higher, than those of the CR and R1, respectively. The contributing factor in the enhanced R2 surface area was their hollow structure with high roughness factor and Pt % utilization (Table 1). The roughness factor (R.F.)^73^, the number of exposed atoms and the utilization percentage of Pt (U_Pt_)^74^ were calculated using following equations:

3 $${\rm{R}}{\rm{.F}}={{\rm{S}}}_{{\rm{ESA}}}/({\rm{apparent}}\,{\rm{surface}}\,{\rm{area}})$$

4 $${{\rm{N}}}_{{\rm{s}}}={{\rm{Q}}}_{{\rm{ct}}}/{{\rm{Q}}}_{{\rm{e}}}$$

5 $${{\rm{U}}}_{{\rm{Pt}}}={{\rm{N}}}_{{\rm{s}}}/{{\rm{N}}}_{{\rm{t}}}$$

**Table 1:** Activity parameters evaluated from CV measured in 1 M H_2_SO_4_ at a scan rate (υ) of 50 mV.s^−1^_._

Catalyst	S_ESA_/(m^2^.g^−1^)	Roughness Factor	No. of exposed Pt atoms (N_s_)/cm^2^	Utilization % of Pt (U_Pt_)
CR	96.4	122.8	1.6 * 10^17^	41
R1	118.2	150.6	1.97 * 10^17^	50
R2	190.8	243	3.2 * 10^17^	81

N_t_ is the total number of Pt atoms in the electrode catalyst. The electrocatalytic behavior of the various synthesized catalysts was investigated for methanol oxidation in a basic medium by CV scanning. Figure 4b demonstrated the behavior of various catalysts towards 1 M methanol in a 1 M KOH medium. All catalysts demonstrated well-defined anodic peaks due to the oxidation of freshly adsorbed methanol, while in the reverse cycle, a small peak appeared due to the removal of carbonaceous intermediate products^71^. A substantial difference in the electroactivity for the oxidation of the methanol was observed. The mass activity of the R2 catalyst was significantly higher than that of other catalysts. The comparison of the electroactivity of the different catalyst for 1 M CH_3_OH is listed in Table 2. The enhanced catalytic activity of R2 compared to CR, R1, and R0 was due to its high electroactive surface area and hollow nanoboxes, which provided more catalytic sites and the confinement of methanol within the nanovessels.

**Table 2:** Activity parameters evaluated from CVs in 1 M CH_3_OH + 1 M KOH on catalysts.

Catalysts	Onset Potential E/V	Peak Potential Ep/V	Peak current/mA.mg^−1^ Pt	Rate constant k_het_/cm.s^−1^ * 10^−5^
CR	−0.65	−0.18	890	6.2
R1	−0.70	−0.11	3450	12
R2	−0.73	−0.08	5245	21.4

### Comparison of the polarization curves

**Figure 5:**
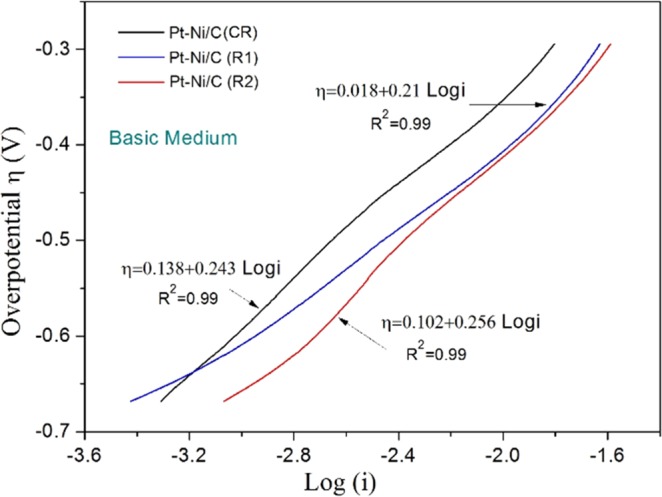
Polarization curves measured in 1 M CH_3_OH + 1 M KOH at a scan rate of 50 mV.s^1^ on Pt-Ni/C(CR), Pt-Ni/C(R1) and Pt-Ni/C(R2) catalysts.

Comparison of polarization plots for the electrooxidation of methanol on both conventional and RME-synthesized catalysts is presented in Fig. 5. The current-potential data were taken by analyzing the forward anodic peaks of 1 M methanol in KOH electrolyte (Fig. 4b). The Tafel Eqs 6 & 7 were used to evaluate the kinetic parameters from the polarization curves^71^:

6 $${\rm{E}}={{\rm{E}}}^{{\rm{0}}}-{\rm{b}}\,\log \,{i}^{{\rm{0}}}+{\rm{b}}\,\log \,I$$

The Tafel equation can be demonstrated as

7 $${\rm{E}}={\rm{a}}+{\rm{b}}\,\log \,{\rm{i}}$$

**Figure 6:**
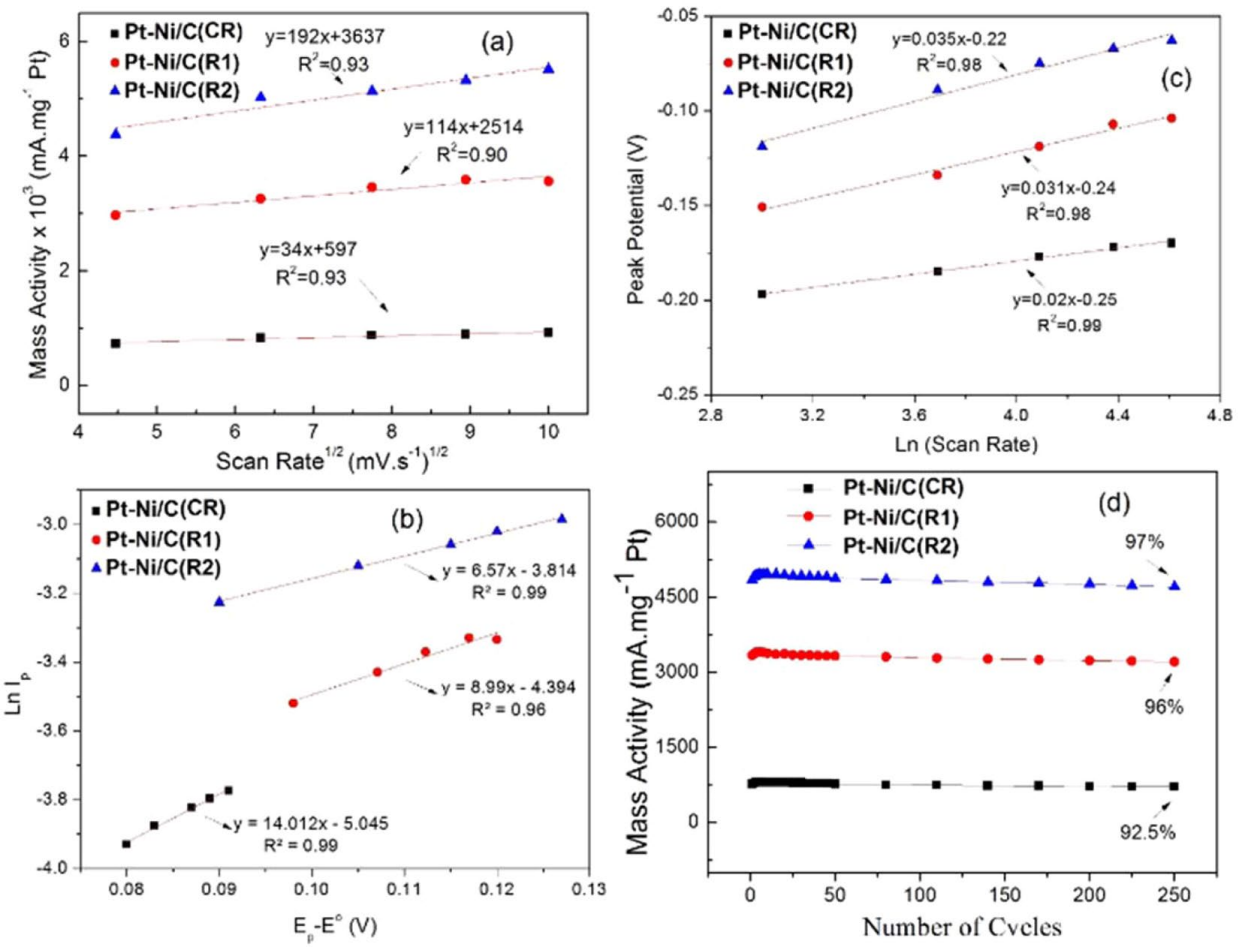
(**a**) Plot of mass activity vs. υ^1/2^, (**b**) plot of Ln(Ip) vs. Ep − Eo (**c**) irreversibility plot Ep vs. Ln(υ) (**d**) Durability study for methanol on Pt-Ni/C (CR) and Pt-Ni/C (RME) in basic media.

The kinetic parameters evaluated from the polarization curves are listed in Table S7. The higher exchange current density means an increased intrinsic rate of electron transfer between the electrolyte and electrode, which indicates higher catalytic kinetics. The values of the product of electron transfer coefficient and a number of electrons involved in the rate-determining step “αn_α_” were calculated from the values of Tafel slopes^75^. The value of α was calculated by the linear relationship of the Ln of scan rate and the peak potential of methanol (Fig. 6c). The number of electrons found with the help of α was in the range of 0.26 to 0.32, which demonstrates the complex nature of methanol oxidation. It is also clear that no more than one electron is involved in the rate-determining step^73^.

### Effect of scan rate on peak current and peak potential of methanol

Methanol oxidation was investigated for CR (Fig. S13a), R1 (Fig. S13b), and R2 (Fig. 4c) using CV at different scan rates. It was observed that both the peak current and peak potential changed with the scan rate. The peak potentials moved towards less negative values with the increase in scan rate, indicating the structural change in the electrochemically formed surface film. Meanwhile, the increase in the peak current with an increase in scan rate indicated progressive enrichment with accessible electroactive species on or near the electrode surface^76^. The peak potential shift and increase in peak current was more pronounced in the Pt-Ni/C-RME catalysts compared to the Pt-Ni/C-CR catalyst, indicating better efficiency of the catalysts synthesized by the RME method.

By applying the Randles-Sevcik equation, linear plots between MA versus ν^1/2^ shown in Fig. 6a suggest that MOR might be a diffusion-controlled process in basic media^77^. For all the scan rates, the mass activity of R2 is higher compared to R1 and CR as shown in the Fig. 6a, and the slope for R2 is almost 5.6 times greater than that of CR and 1.7 times greater than that of R1. This improvement could be attributed to the higher active surface area of the Pt catalysts per unit geometric area of the electrode.

As discussed above, a peak shift was observed as the scan rate increased. A linear relationship was observed between Ep and Ln(υ) (Fig. 6c)^77^. The value of α, calculated from the slope of the plot for CR, R1 and R2, was 0.75, 0.86 and 0.88, respectively, and is comparable to the reported values^78^. For total irreversibility of the electron transfer process, the slope of E_p_ vs. Ln(υ) should be greater than 30 mV per decade and the width of the CV curves (E_p_- E_p/2_) should be significantly greater than 60 mV^79^. The electrochemical reaction of methanol on R1 and R2 was an irreversible electron transfer process, as inferred by their CV curves.

### Evaluation of heterogeneous rate constant

For nanocatalysis, the rate of reaction depends on the shape, size, and activity of the nanocatalyst material along with other common factors^26^. Heterogeneous rate constants (k_het_) for the MO at temperature T can be determined from the intercept by plotting the graph of (E_p_ − E°) and ln(I_p_) (Fig. 6b) using the Nicholson–Shain^41^ Eq. 8,

8 $${i}_{{\rm{p}}}={\rm{0.227}}\,{{\rm{nFAC}}}_{{\rm{o}}}{{\rm{k}}}_{{\rm{het}}}.\exp [({{\rm{\alpha }}{\rm{n}}}_{{\rm{\alpha }}}.{\rm{F}}/{\rm{RT}})({{\rm{E}}}_{{\rm{p}}}-E^\circ )]$$

In this equation F and R are the usual constants, A cm^2^ is the surface area of the electrode, C_o_ mol.cm^−3^ is the methanol concentration, and n is the total number of electrons transferred in the process (n = 6 for methanol). E° is the potential at 0.85 *i*_p_, while αn_α_ is considered one parameter, namely, the product of the electron transfer coefficient (α) and n_α_, the number of electrons involved in the rate-determining step. The kinetic study showed that the methanol reaction on the surface of R2 is 1.8 times faster compared to R1 and 3.5 faster compared to CR (Table 2). Despite the same composition of Pt-Ni in R1 and R2, the R2 displayed a better electrochemical activity. The enhanced activity suggests that the cavity of the hollow nanobox participated in the electrochemical activity, which improved the electrochemical surface area and the reaction of the R2 catalyst, and it is most likely a result of the confinement effect of the reactions occurring within the cavity. In a nanocavity, atoms on the corners are better protected than those on the faces of the nanoboxes and are thus resistant to dissolution and agglomeration. R2 shows much better mass activity than the reported Pt based nanomaterials such as Pt-Bi/GNs (2005 mA/mg Pt)^80^, Pt/sG (PVA) (2250 mA/mg Pt)^81^, Pt-Au/RGO (1380 mA/mg Pt)^82^. MA of R2 is slightly less than recently reported Pt-Ag/G (5628 mA/mg Pt)^83^, however, in this case total metals content is 33% while in our study 14.3% which shows the superior nature of R2.

Great focus has been paid to the durability of the catalyst in order to commercialize it in DMFCs. The bi-functional mechanism, comprising the incorporation of a non-noble metal to replace the Pt catalyst, improved the durability of catalysts. Therefore, the durability depends upon the mechanism of the MO occurring at the catalyst surface. Figure 6d shows the durability of catalysts in term of the mass activity obtained for 1 M CH_3_OH + 1 M KOH at a scan rate 50 mV.s^**−**1^ for 250 cycles in a single run. MA increases in the initial few cycles and then becomes almost constant. R2 retained almost 97% while CR retained 92.5%. The increased stability of R2 and R1 is due to the improved surface morphology, resistance to aggregation and better regeneration of the poisoned Pt atoms^84^.

## Conclusion

Efficient Pt-Ni hollow nanoboxes are introduced for the efficient electrooxidation of methanol in fuel cells. The catalyst was synthesized with an improved RME method. The modified RME method was a facile and highly efficient method for the synthesis of hollow nanostructures having precise compositions, and the method contributed in two ways: by improving the particle morphology and increasing the intimate contact between Ni and Pt NPs, thus increasing the supplying of oxygenic species to the Ni to Pt sites. The cavity of the hollow nanoparticles most likely exerted a confinement effect on the chemical reaction occurring in the cavity. The catalysts synthesized through the RME method not only showed higher activity but also better stability. One main reason for the bad performance of the conventional reduction method is the difference between the theoretical and experimental ratios of Pt and Ni, which were confirmed by DFT. As demonstrated by the DFT studies, the RME method provided a precise composition of Pt-Ni catalyst manage the CO poising. The synthesized Pt-Ni/C (R2) hollow nanoboxes may be an efficient catalyst material for the oxidation of methanol due to its unique morphology, large surface area, high stability and low cost.

## Supplementary information

Supplementary information
