# Supplementary material for: Synthesis of Hollow Pt-Ni Nanoboxes for Highly Efficient Methanol Oxidation
Source: Sci Rep. 2019 Oct 24;9:15273. doi: 10.1038/s41598-019-51780-y (PMC6813309; doi:10.1038/s41598-019-51780-y)
Supplement: Supplementary file 1 — Supplementary information [file 41598_2019_51780_MOESM1_ESM.docx]

Synthesis of Hollow Pt-Ni Nanoboxes for Highly Efficient Methanol Oxidation

Rabia Jamil,*^a^ Manzar Sohail*^a^, Nadeem Baig^b^ , Muhammad S. Ansari^c^, Riaz Ahmed^c^

*^a^Department of Chemistry, School of Natural Sciences, National University of Science and Technology (NUST), Islamabad 44000, Pakistan.*

*^b^Chemistry Department, King Fahd University of Petroleum and Minerals, Dhahran 31261, Saudi Arabia.*

*^c^Department of Chemistry, Quaid-i-Azam University Islamabad 45320, Pakistan.*

**Supporting Information**

**Table S1.** Components of Reverse Microemulsion (RME) in Simultaneous Reaction Procedure

| RME (I) | RME (II) |
| --- | --- |
| 25mL of hexane  12.5mL of TX-100  25mL of *n*-hexanol  3mL of 0.03M PtCl_4_ + 1.02 mL of 0.07M NiCl_2_.6H_2_O | 25mL of hexane  12.5mL of TX-100  25mL of *n*-hexanol  4 mL 1.4 M NaBH_4_ |

**Table S2.** Components of Reverse Microemulsions in Sequential Reaction Procedure

| RME(I) | RME(II) | RME(III) | RME(IV) |
| --- | --- | --- | --- |
| 6.25mL of hexane  3.12mL of TX-100  6.25mL of *n*-hexanol  1mL of 0.07M NiCl_2_.6H_2_O | 12.5mL of hexane  6.2mL of TX-100  12.5mL of *n*-hexanol  2 mL 1.4 M NaBH_4_ | 19mL of hexane  9.4mL of TX-100  19mL of *n*-hexanol  3.02mL of 0.03M PtCl_4_ | 12.5mL of hexane  6.2mL of TX-100  12.5mL of *n*-hexanol  2 mL 1.4 M NaBH_4_ |


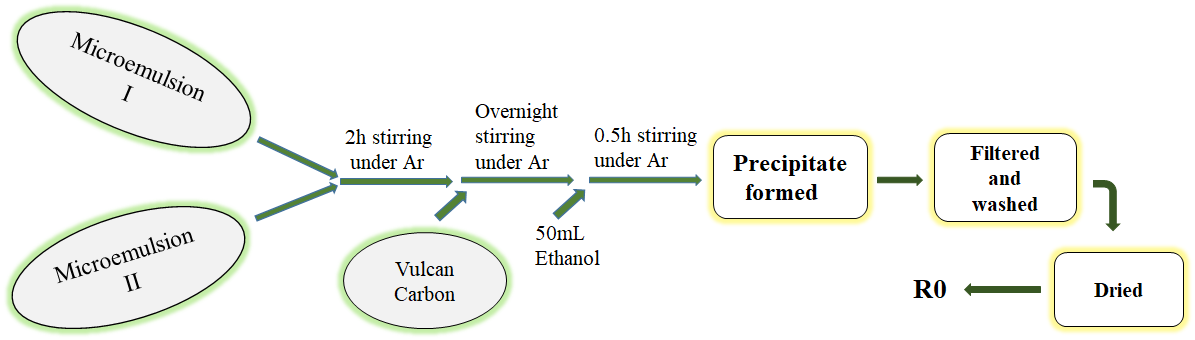


**Figure S1.** Simultaneous reaction procedure


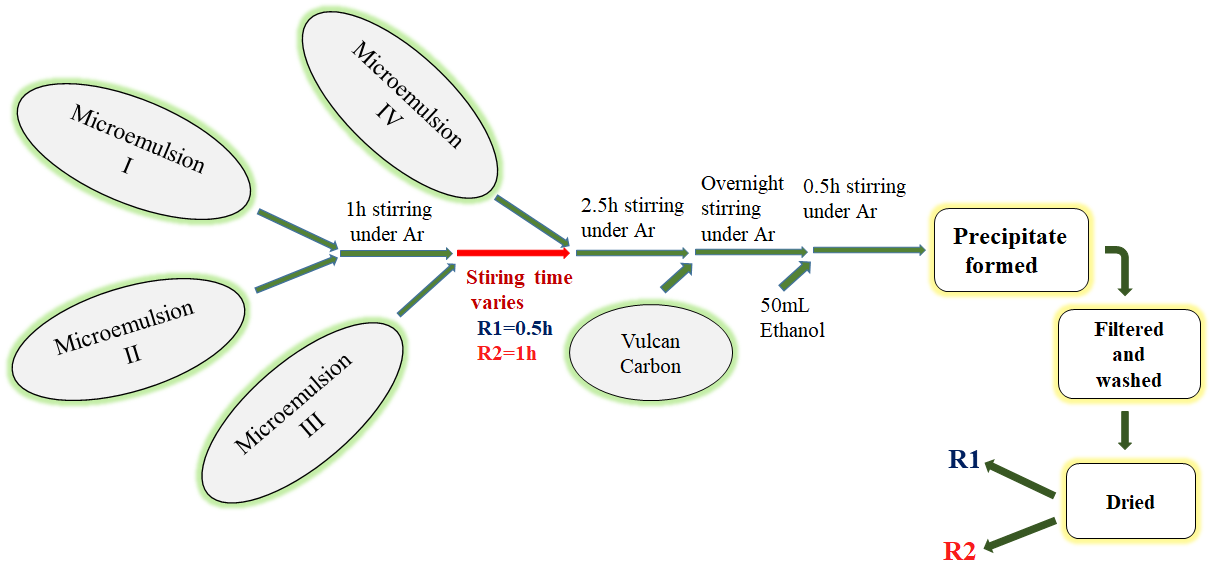


**Figure S2.** Sequential reaction procedure


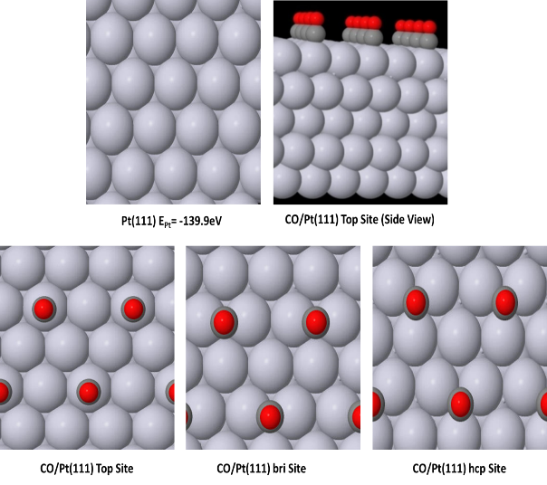


**Figure S3.** Overview of Adsorbate structures of CO on different Pt(111) surfaces


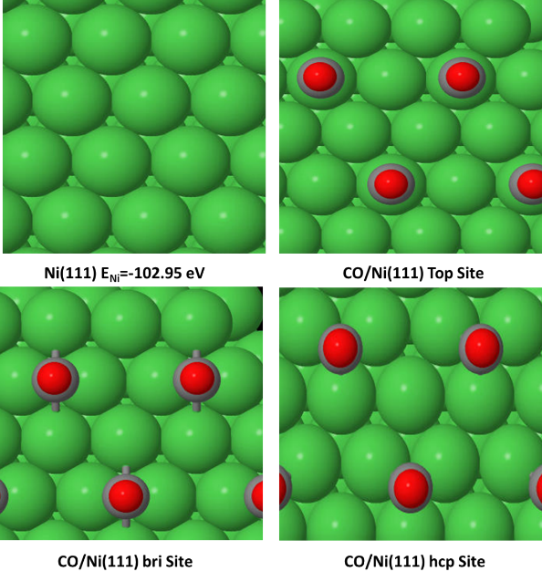


**Figure S4.** Overview of Adsorbate structures of CO on different Ni(111) surfaces


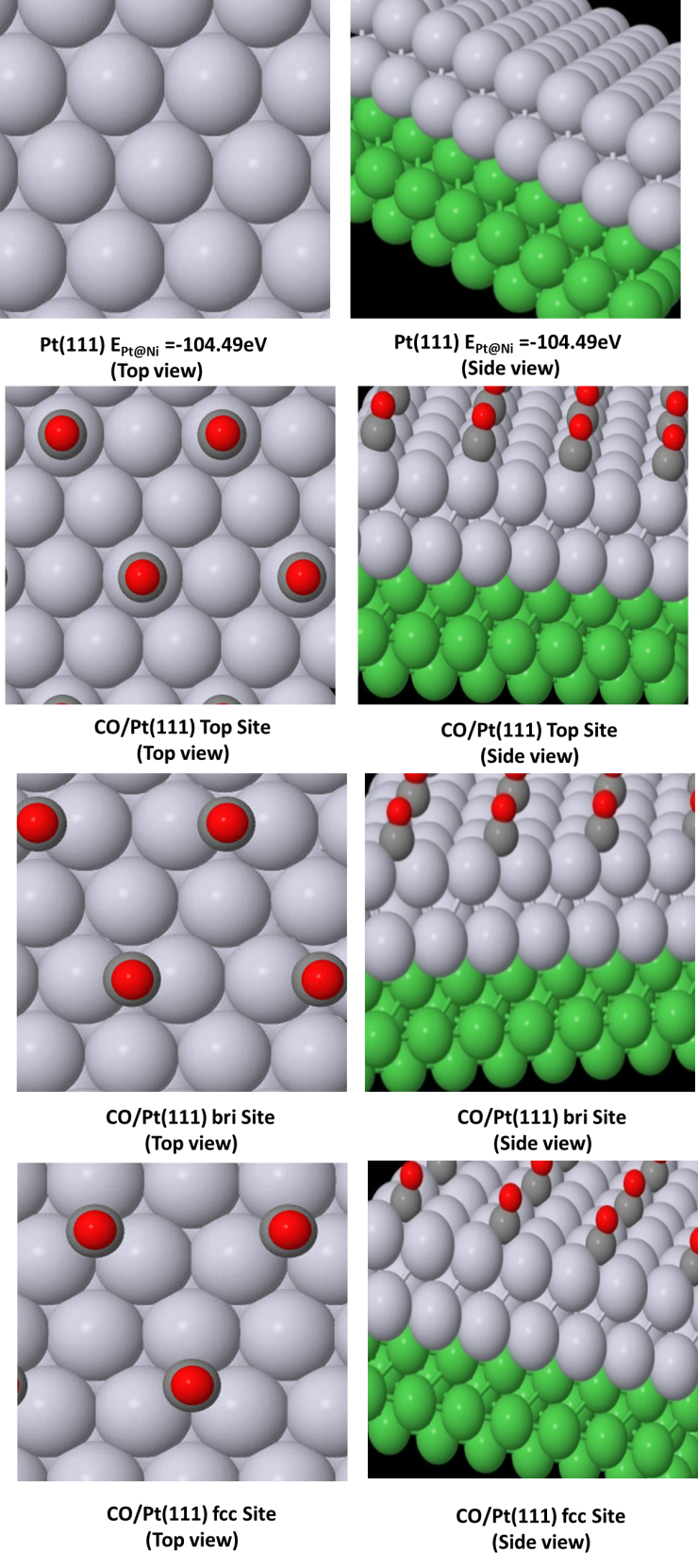


**Figure S5.** Overview of Adsorbate structures of CO on different Pt(111) surfaces (Slab containing top two Pt layers and bottom 3 layers of Ni).

**Table S3:** Summary of DFT calculations results: CO adsorption energy (E_ads_/eV) and bond length of CO (D_C-O_/ Å)

| **Surface** | **Unit cell** | **Adsorption site** | **E_ads_/eV** | **D_C-O_/A^o^** |
| --- | --- | --- | --- | --- |
| **Pt (111)** | P(2x2) | top | -1.48 | 1.14 |
|  |  | bri | -1.59 | 1.14 |
|  |  | hcp | -1.64 | 1.14 |
| **Ni (111)** | P(2x2) | top | -1.73 | 1.19 |
|  |  | bri | -2.03 | 1.18 |
|  |  | hcp | -2.15 | 1.18 |
| **P_1_Ni_3_** | P(2x2) | top | -1.62 | 1.16 |
|  |  | bri | -1.46 | 1.17 |
|  |  | hcp | -1.81 | 1.19 |
| **Pt_1_Ni_1_** | P(2x2) | top | -1.42 | 1.16 |
|  |  | bri | -1.44 | 1.18 |
|  |  | hcp | -1.43 | 1.18 |
| **Pt_3_Ni_1_** | P(2x2) | top | -1.52 | 1.16 |
|  |  | bri | -1.54 | 1.18 |
|  |  | hcp | -1.53 | 1.18 |
| **Pt@Ni** | P(2x2) | top | -1.43 | 1.18 |
|  |  | bri | -1.61 | 1.18 |
|  |  | fcc | -1.7 | 1.19 |

**Figure S6.** FTIR spectrum of functionalized VC shows presence of (C=O, –OH) groups

**Table S 4:** XRD parameters evaluated for Standard Pt, CR, R1 and R2

| **Catalysts** | **Struct-ures** | **Position 2θ**  **(degree)** | ***d*-spacing Å** | **Lattice param-eter**  ɑ **/ Å** | **Cell volume V / Å^3^** | **Density ρ_X-ray_ /g ^.^cm^-3^** | **Avg.**  **Crystall-ite size D/nm** | **Crystallin-ity** | **Pt-Pt bond length/ Å** | **Chemical surface area/m^2..^g^-1^** |
| --- | --- | --- | --- | --- | --- | --- | --- | --- | --- | --- |
| Pt Standard | cubic | 39.67 | 2.27 | 3.912 | 59.87 | 21.5 | - | - | 2.77 | - |
| CR | cubic | 40.07 | 2.25 | 3.897 | 59.18 | 18.4 | 14.3 | 0.041 | 2.76 | 19.6 |
| R1 | cubic | 40.52 | 2.23 | 3.856 | 57.33 | 19.0 | 4.5 | 0.028 | 2.73 | 62.6 |
| R2 | cubic | 40.82 | 2.21 | 3.829 | 56.14 | 19.4 | 5.1 | 0.029 | 2.71 | 54.8 |

Relative cystallinity of Pt particles= intensity of Pt(111)/intensity of C(002) [^1^](#_ENREF_1)

Pt-Ptbond length=ɑ/$\sqrt{2}$ (ɑ is lattice parameter)

Chemical surface area ($SCSA$)

$$SCSA=\frac{6x{10}^{3}}{\rho D}$$

Ρ is Pt density 21.4g/cm^3^, D is the particle size calculated through XRD [^2^](#_ENREF_2)


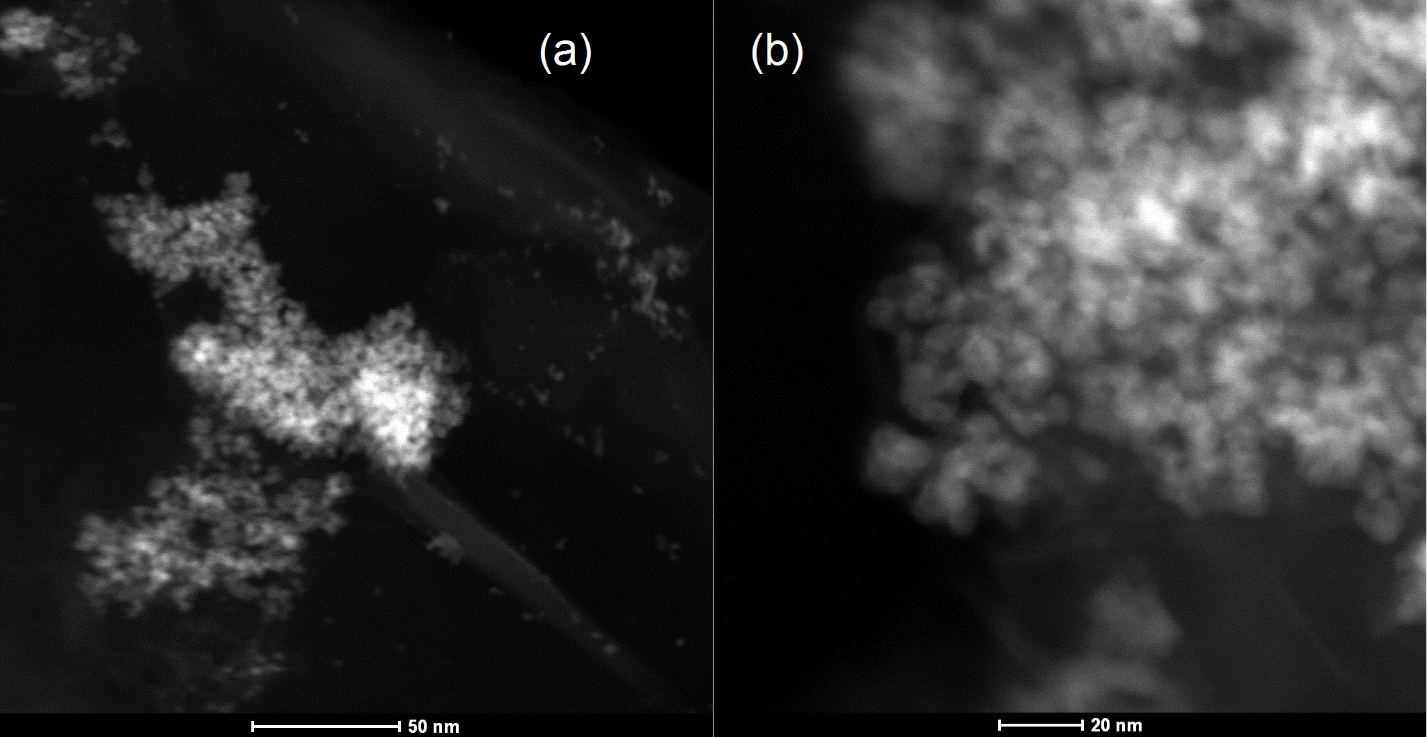


**Figure S7A.** STEM-HAADF image at (a) 50 nm and (b) 20 nm of Pt-Ni/C(CR).


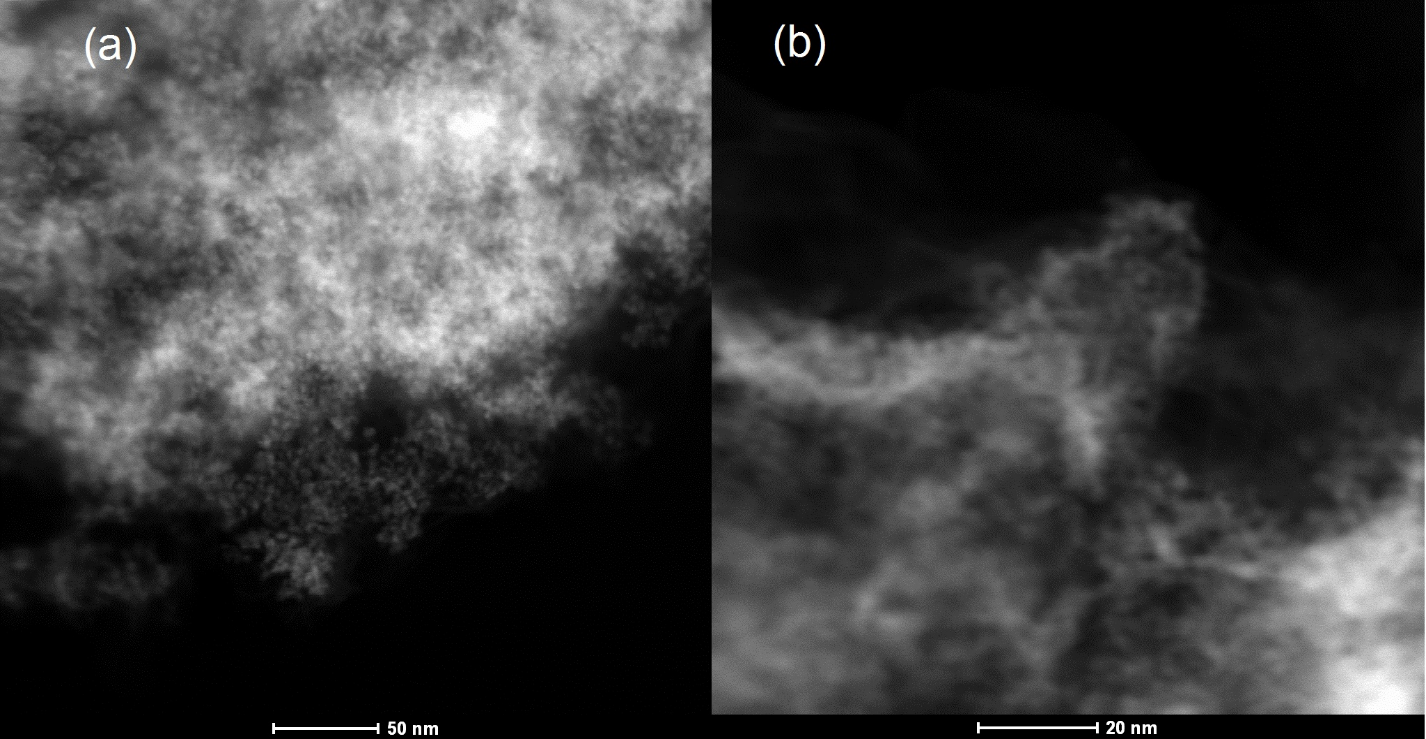


**Figure S7B.** STEM-HAADF image at (a) 50 nm and (b) 20 nm of Pt-Ni/C(R0).


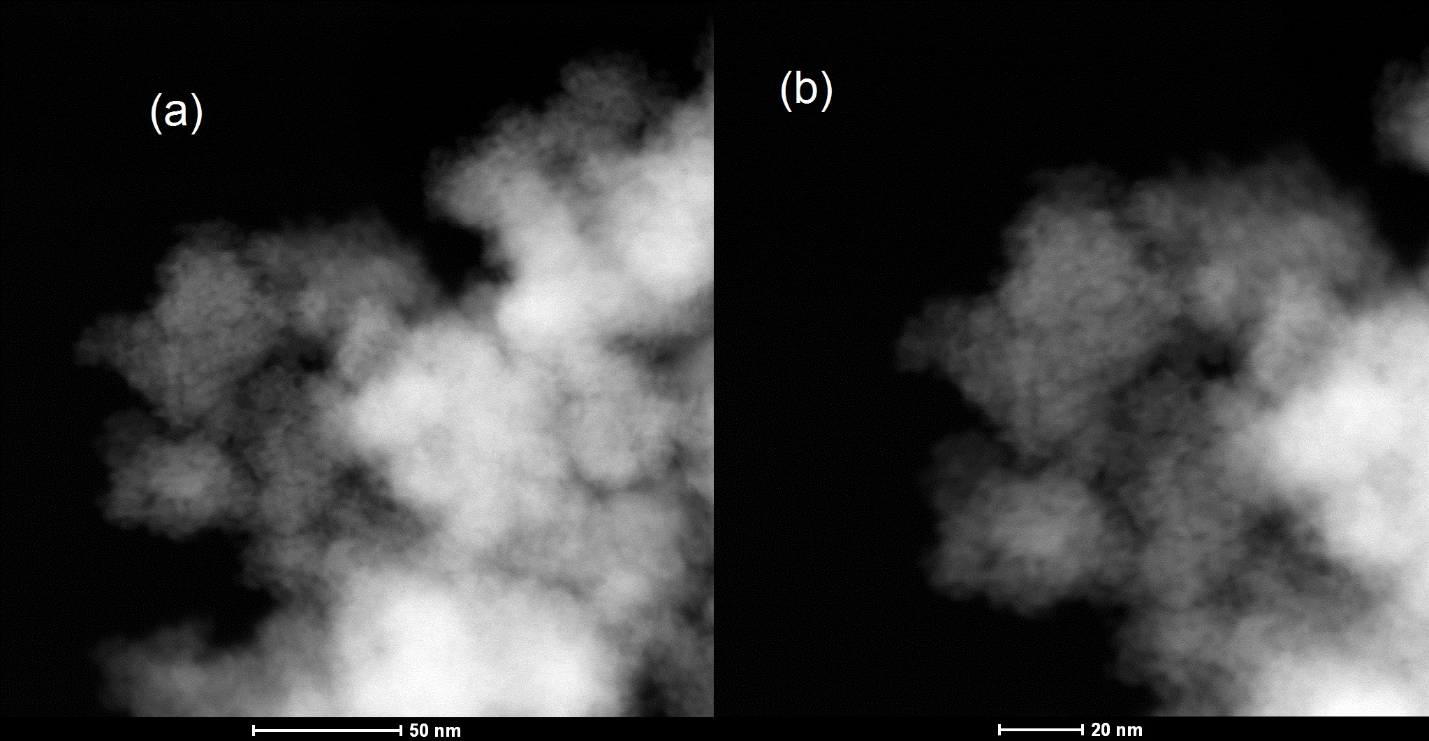


**Figure S7C.** STEM-HAADF image at (a) 50 nm and (b) 20 nm of Pt-Ni/C(R1).


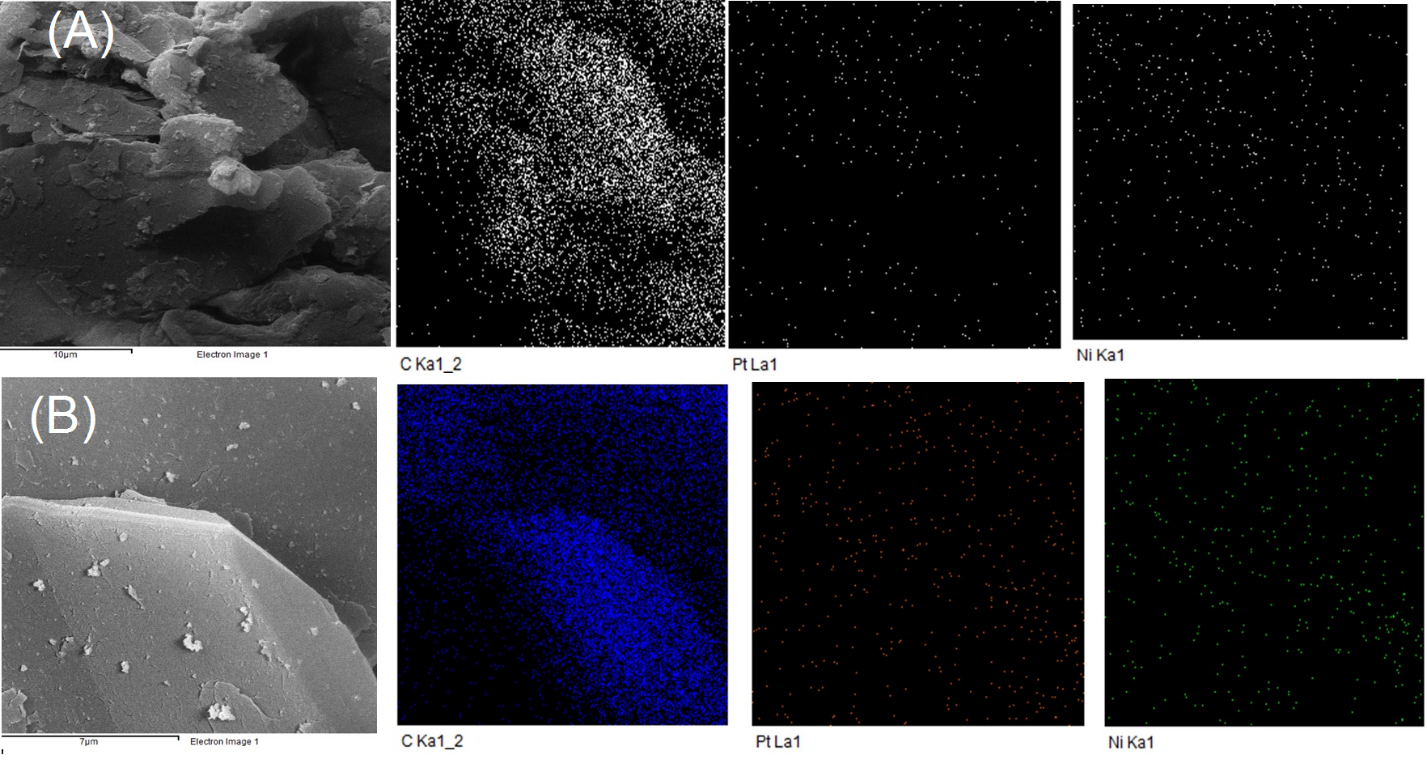


**Figure S8.** Elemental mapping of Pt-Ni/C synthesized by (A) conventional reaction(CR) and (B) Reverse emulsion method (R1)


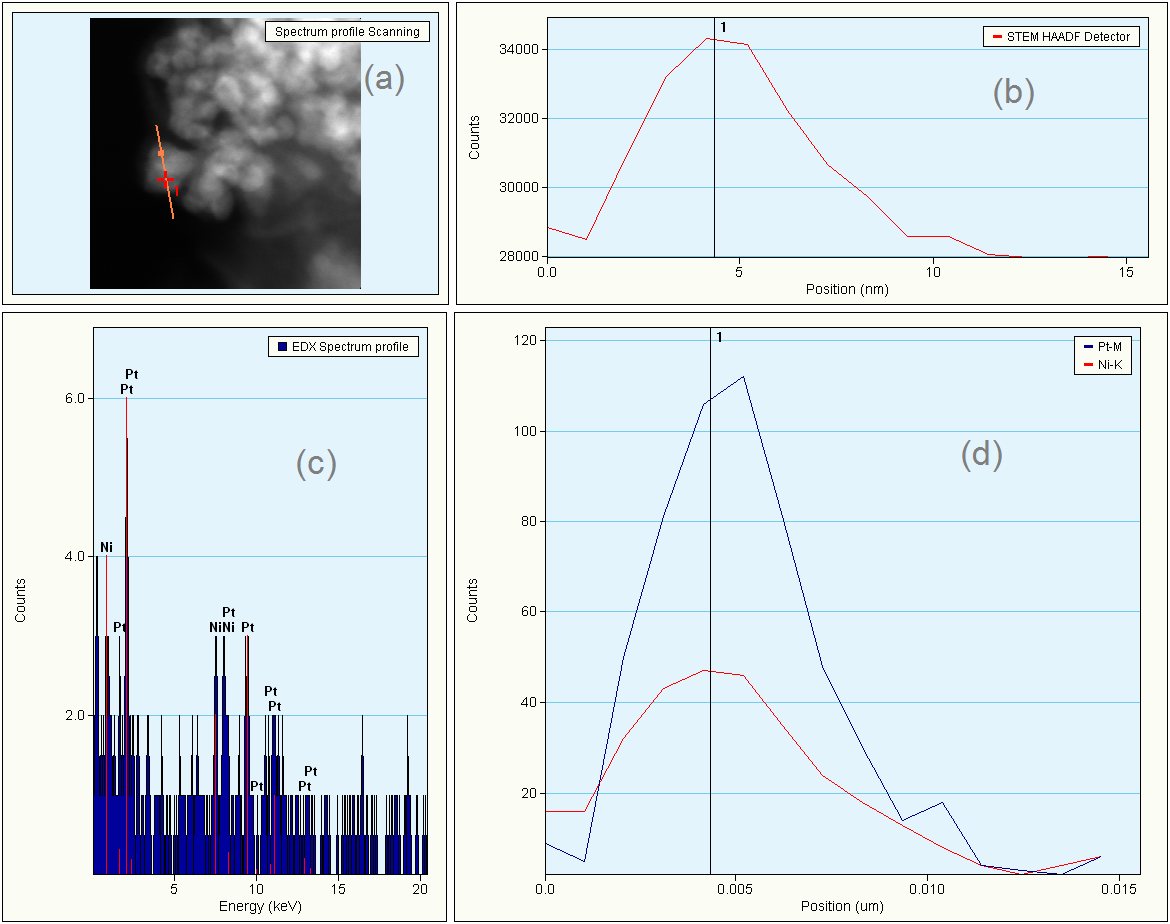


**Figure S9.** (a) HAADF-STEM of CR; (b) STEM HAADF detector, (c) EDX area scan along the square; (d) EDX line scan along the arrow


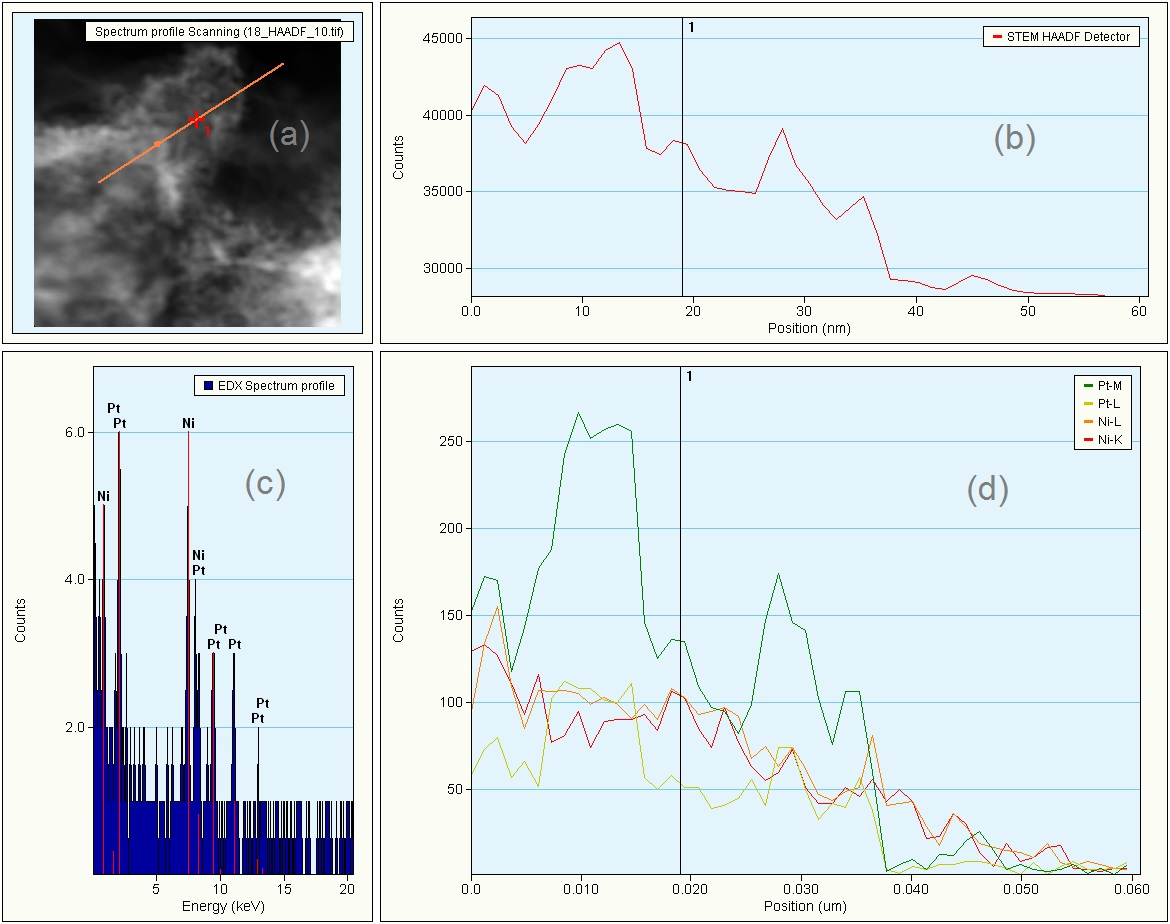


**Figure S10.** (a) HAADF-STEM of Pt-Ni/C (R0); (b) STEM HAADF detector, (c) EDX area scan along the square; (d) EDX line scan along the arrow

**
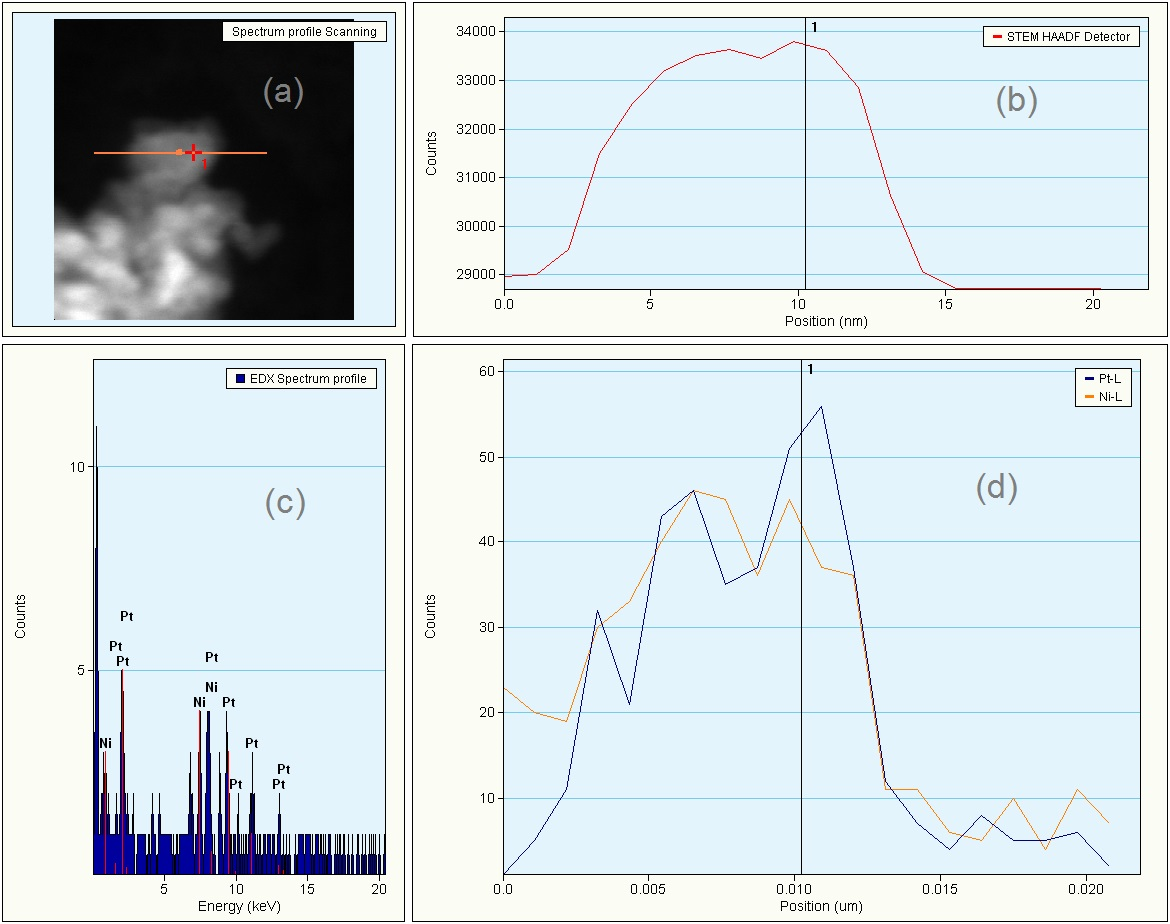
**

**Figure S11.** (a) HAADF-STEM of R1; (b) STEM HAADF detector, (c) EDX area scan along the square; (d) EDX line scan along the arrow.

**Figure S12.** CVs measured in 1 M CH3OH + 1 M KOH at scan rate 50 mV.s^-1^ on Pt-Ni/C (R0)

**
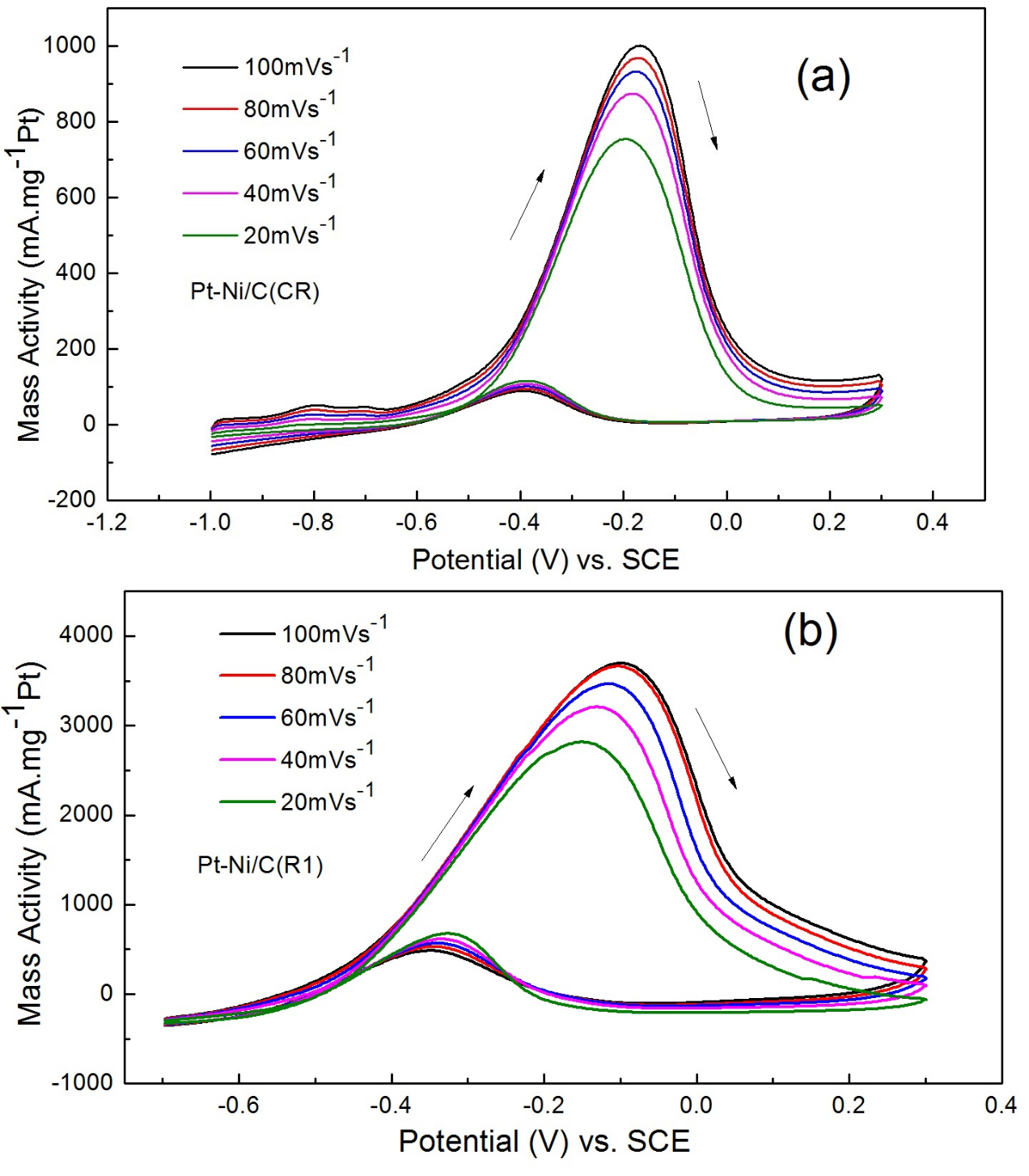
**

**Figure S13.** CVs measured in 1 M CH_3_OH + 1 M KOH at different scan rates on (a) Pt-Ni/C-CR, (b) Pt-Ni/C-R1


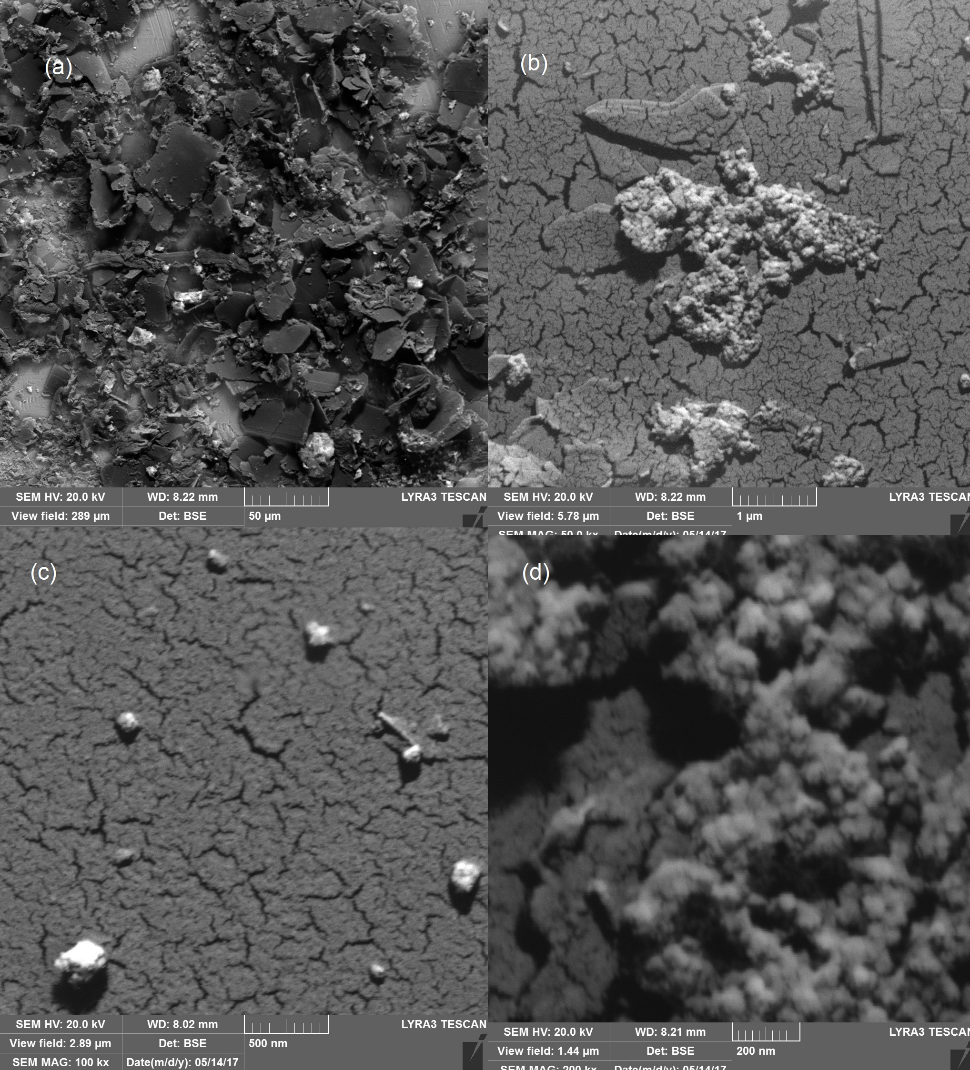


**Figure S14.** SEM images of Pt-Ni/C-R2 catalysts at different resolutions.


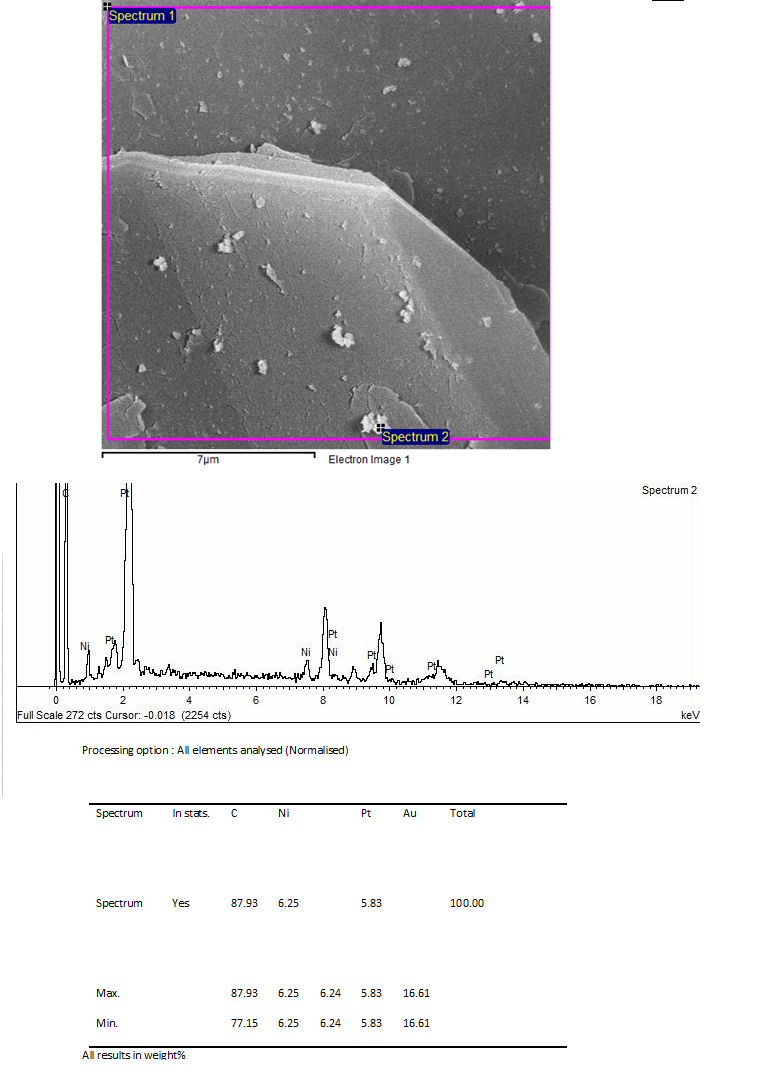


Figure S15. EDX spectra of the of Pt-Ni/C-R2 catalyst.

**Table** S5: Composition of the catalyst determined by theoretical and EDX analysis

| **Catalysts** | **Theoretical Calculations** | | **Experimental composition**  **(EDX )** | | **Composition wt. %** | | | **Metal loading wt. %** |
| --- | --- | --- | --- | --- | --- | --- | --- | --- |
|  | **Mass Ratio Pt:Ni** | **Atomic Ratio Pt:Ni** | **Mass Ratio Pt:Ni** | **Atomic Ratio Pt:Ni** | **Pt** | **Ni** | **C** |  |
| **Pt-Ni/C (CR)** | 3.34:1 | 1:1 | 6.3:1 | 2:1 | 17.1 | 2.7 | 80.2 | 19.8 |
| **Pt-Ni/C (R1)** | 3.34:1 | 1:1 | 3.6:1 | 1:1 | 11.4 | 3.19 | 85.4 | 14.6 |
| **Pt-Ni/C (R2)** | 3.34:1 | 1:1 | 3.4:1 | 1:1 | 11.1 | 3.2 | 85.7 | 14.3 |

**Table S 6:** Activity parameters evaluated from CVs in 1M CH_3_OH + 1M KOH on Pt-Ni/C (R0)

| **Catalysts** | **Onset Potential E/V** | **Peak Potential E_p_/V** | **Mass activity /mA.mg^-1^** |
| --- | --- | --- | --- |
| Pt-Ni/C(R0) | -0.69 | -0.16 | 2870 |

**Table S7:** Polarization data evaluated from Tafel plots in 1M CH_3_OH + 1KOH

| **Catalyst/C** | **Tafel’s slope “b”**  **(V decade^-1^)** | **Intercept of E**  **vs Log i** | **αn_α_** | ί^o^/  **mA.mg^-1^ Pt** |
| --- | --- | --- | --- | --- |
| Pt-Ni (CR) | 0.243 | 0.14 | 0.24 | 960 |
| Pt-Ni (R1) | 0.21 | 0.02 | 0.28 | 3510 |
| Pt-Ni (R2) | 0.256 | 0.10 | 0.23 | 5600 |

| S. No. | Catalyst  (Nanostructures) | Onset Potential / V | E_p_ vs RHE / V | Medium | Current density | Catalytic properties | Reference |
| --- | --- | --- | --- | --- | --- | --- | --- |
| 1 | Pt/C/graphene | 0.4 | 0.6 | Acidic | 424.6 mA.mg-1 Pt | Good electrocatalytic activities for both methanol and ethanol but CO poisoning occurs. | [^3^](#_ENREF_3) |
| 2 | PtRuNi/MWCNT | 0.02 | 0.6 | acidic | 4000 mA.mg-1 Pt | Very high MOR current density with improved CO poisioning | [^4^](#_ENREF_4) |
| 3 | PtNi/C | 0.45 | 0.7 | Alkaline | 4.9 mA.cm^-2^ | Improvement in catalytic activity with Ni coating. CO poisoning decreased by OH adsorption. | [^5^](#_ENREF_5) |
| 4 | PtNi/vulcan C | 0.28 | 0.4 | acidic | 12 mA.mg-1 Pt | Slight improvement in activity compared to only Pt/C | [^6^](#_ENREF_6) |
| 5 | Pt-Ni branched nanocrystals | 0.38 | 0.6 | acidic | - | Catalytic activity and stability improved, compared to only Pt | [^7^](#_ENREF_7) |
| 6 | Pt/Ni(OH)2/rGO | 0.4 | 0.78 | Alkaline | 1236  mA.mg^-1^  Pt | High catalytic activity with very good stability. After 80,000 s can sustain more than 400 mA.mg^-1^ Pt current density. | [^8^](#_ENREF_8) |
| 7 | Pt–Ni/C encapsulated in polyaniline shells | 0.5 | 0.9 | Alkaline | 2500  mA.mg^-1^  Pt | High catalytic activity and increased durability due to polyaniline shells | [^9^](#_ENREF_9) |
| 8 | Pt–Ni–P nanocages | 0.4 | 0.65 | Acidic | 1220 mA.mg^-1^  Pt | Good catalytic activity and durability owing to effective charge transfer | [^10^](#_ENREF_10) |
| 9 | Pt-Ni dumbbells | 0.4 | 0.6 | Acidic | 3020  mA.mg^-1^  Pt | Very high catalytic activity and retained 82 % of the activity after 1000 CV cycles | [^11^](#_ENREF_11) |
| 10 | Pt-Ni nanoboxes | 0.27 | 0.92 | Alkaline | 5245 mA.mg^-1^  Pt | Very high catalytic activity due to nano-confinement effect and 92.5 % of the activity was retained after 250 CV cycles. | This work |

**References**

1 Wang, R., Li, H., Feng, H., Wang, H. & Lei, Z. Preparation of carbon-supported core@shell PdCu@PtRu nanoparticles for methanol oxidation. *J. Power Sources* **195**, 1099-1102, doi:https://doi.org/10.1016/j.jpowsour.2009.08.055 (2010).

2 Qian, Y. *et al.* PtM/C Catalyst Prepared Using Reverse Micelle Method for Oxygen Reduction Reaction in PEM Fuel Cells. *J. Phys. Chem. C* **112**, 1146-1157, doi:10.1021/jp074929i (2008).

3 Zhao, L. *et al.* Hybrid of carbon-supported Pt nanoparticles and three dimensional graphene aerogel as high stable electrocatalyst for methanol electrooxidation. *Electrochim. Acta* **189**, 175-183, doi:https://doi.org/10.1016/j.electacta.2015.12.072 (2016).

4 Zhao, Y., Fan, L., Ren, J. & Hong, B. Electrodeposition of Pt–Ru and Pt–Ru–Ni nanoclusters on multi-walled carbon nanotubes for direct methanol fuel cell. *Int. J. Hydrogen Energy* **39**, 4544-4557, doi:https://doi.org/10.1016/j.ijhydene.2013.12.202 (2014).

5 Lu, S., Li, H., Sun, J. & Zhuang, Z. Promoting the methanol oxidation catalytic activity by introducing surface nickel on platinum nanoparticles. *Nano Research* **11**, 2058-2068, doi:10.1007/s12274-017-1822-x (2018).

6 Amin, R. S., Abdel Hameed, R. M., El-Khatib, K. M. & Elsayed Youssef, M. Electrocatalytic activity of nanostructured Ni and Pd–Ni on Vulcan XC-72R carbon black for methanol oxidation in alkaline medium. *Int. J. Hydrogen Energy* **39**, 2026-2041, doi:https://doi.org/10.1016/j.ijhydene.2013.11.033 (2014).

7 Niu, Z., Wang, D., Yu, R., Peng, Q. & Li, Y. Highly branched Pt–Ni nanocrystals enclosed by stepped surface for methanol oxidation. *Chemical Science* **3**, 1925-1929, doi:10.1039/C2SC00004K (2012).

8 Huang, W. *et al.* Highly active and durable methanol oxidation electrocatalyst based on the synergy of platinum–nickel hydroxide–graphene. *Nature Communications* **6**, 10035, doi:10.1038/ncomms10035

https://[www.nature.com/articles/ncomms10035#supplementary-information](http://www.nature.com/articles/ncomms10035#supplementary-information) (2015).

9 Kim, K. S., Hong, Y., Kim, H. C., Choi, S.-I. & Hong, J. W. Ultrathin-Polyaniline-Coated Pt–Ni Alloy Nanooctahedra for the Electrochemical Methanol Oxidation Reaction. *Chemistry – A European Journal* **25**, 7185-7190, doi:10.1002/chem.201900238 (2019).

10 Deng, K. *et al.* Pt–Ni–P nanocages with surface porosity as efficient bifunctional electrocatalysts for oxygen reduction and methanol oxidation. *Journal of Materials Chemistry A* **7**, 9791-9797, doi:10.1039/C9TA00928K (2019).

11 Gong, W. *et al.* Cross-double dumbbell-like Pt–Ni nanostructures with enhanced catalytic performance toward the reactions of oxygen reduction and methanol oxidation. *Applied Catalysis B: Environmental* **246**, 277-283, doi:https://doi.org/10.1016/j.apcatb.2019.01.061 (2019).
